# Mitochondrial Membrane Intracellular Communication in Healthy and Diseased Myocardium

**DOI:** 10.3389/fcell.2020.609241

**Published:** 2020-12-23

**Authors:** Vishnu K. Kumar, Atreju Lackey, Jonathan Snyder, Sunil Karhadkar, Ajay D. Rao, Antonio DiCarlo, Priscila Y. Sato

**Affiliations:** ^1^Department of Pharmacology and Physiology, Drexel University College of Medicine, Philadelphia, PA, United States; ^2^Department of Surgery, Temple University Lewis Katz School of Medicine, Philadelphia, PA, United States; ^3^Section of Endocrinology, Diabetes and Metabolism, Temple University Lewis Katz School of Medicine, Philadelphia, PA, United States; ^4^Center for Metabolic Disease Research, Temple University Lewis Katz School of Medicine, Philadelphia, PA, United States

**Keywords:** mitochondria, endoplasmic reticulum stress, heart, mitochondria-ER communication, membrane communication mechanism

## Abstract

Research efforts in the twenty-first century have been paramount to the discovery and development of novel pharmacological treatments in a variety of diseases resulting in improved life expectancy. Yet, cardiac disease remains a leading cause of morbidity and mortality worldwide. Over time, there has been an expansion in conditions such as atrial fibrillation (AF) and heart failure (HF). Although past research has elucidated specific pathways that participate in the development of distinct cardiac pathologies, the exact mechanisms of action leading to disease remain to be fully characterized. Protein turnover and cellular bioenergetics are integral components of cardiac diseases, highlighting the importance of mitochondria and endoplasmic reticulum (ER) in driving cellular homeostasis. More specifically, the interactions between mitochondria and ER are crucial to calcium signaling, apoptosis induction, autophagy, and lipid biosynthesis. Here, we summarize mitochondrial and ER functions and physical interactions in healthy physiological states. We then transition to perturbations that occur in response to pathophysiological challenges and how this alters mitochondrial–ER and other intracellular organelle interactions. Finally, we discuss lifestyle interventions and innovative therapeutic targets that may be used to restore beneficial mitochondrial and ER interactions, thereby improving cardiac function.

## Introduction

Mitochondria are paramount for energy production in all tissues, especially in highly energetic organs such as the heart. In the myocardium, mitochondria compose around 30% of the total cell volume and synthesize 6 kg of ATP per day via oxidative phosphorylation (Vásquez-Trincado et al., 2016). Mitochondria also have extensive interactive networks with many organelles within the cell, most notably the endoplasmic reticulum (ER). Through these interactions, the mitochondrion also influence Ca^2+^ signaling, reactive oxygen species (ROS), and cell death (Szymański et al., 2017).

Although cardiac disease death rate since the 1950s has substantially declined, it is still the leading cause of mortality among American adults (National Center for Health Statistics, 2018). According to the 2018 National Health Interview Survey, 11.2% of adults in the United States are affected by cardiac diseases, with heart failure (HF) being the most common diagnosis (National Center for Health Statistics, 2018). In 2014, there were 1.1 million emergency department visits with HF as the primary cause and 4.1 million visits with HF as comorbidity. The direct medical costs of HF in 2012 were estimated to be $30.7 billion, with a projected increase to $69.7 billion by 2030 (Jackson et al., 2018). Globally, in 2017, cardiovascular disease was indicated as the cause of nearly 18 million deaths contributing to approximately 330 million years of life lost. These deaths represent a 21.1% increase over the previous decade (Roth et al., 2018).

The expansion of cardiac disease burden is partly due to increased life expectancy and a partial understanding of contributing factors that promote cardiac pathology. Despite the significant amount of research being performed in this area, treatment strategies for many types of cardiac diseases, including HF, remain limited. This review will discuss mitochondrial dynamics, mitochondria and ER roles in cardiac physiology, and therapeutic interventions that focus on mitochondrial and ER stress.

## Importance of Mitochondrial Dynamics, Ultrastructure, and Morphology in Cardiac Physiology

The mitochondrion is a double membrane organelle composed of an outer membrane, intermembrane space, inner membrane, and matrix. The inner mitochondrial membrane exhibits complex folding to increase functional surface area confined within the outer mitochondrial membrane. The electron transport chain, ATP synthase, and many other mitochondrial membrane proteins are localized within the inner mitochondrial membrane (Zick et al., 2009). In yeast and certain mammalian cells, ER tubules constrict the mitochondria prior to the recruitment of proteins involved in mitochondria dynamics (Friedman et al., 2011). Unlike non-cardiac mitochondria, mitochondria in the adult heart are partially immobile, possessing restricted capacity to move and distribute in cytoplasmic tubular networks (Ong et al., 2017). Indeed, continuous mitochondrial networks can span the length of the cell both in the transverse and longitudinal directions. These networks function to exchange cristae components and substrates (Franzini-Armstrong, 2007; Picard et al., 2015). The presence of mitochondrial nanotunnels in cardiomyocytes, but not in skeletal muscle, suggests a direct mode of communication between cardiac mitochondria across distances up to several microns. These nanotunnels contain an outer membrane as well as cristae and therefore allow the sharing of matrix components (Huang et al., 2013; Eisner et al., 2017; Lavorato et al., 2017). Due to limited mitochondrial movement in the myocardium, this mode of communication has been implicated in ensuring content sharing in the absence of proximal direct contact. These structures have been visualized both by live-cell confocal imaging (Huang et al., 2013) and electron microscopy (Lavorato et al., 2017). Indeed, cardiac nanotunnels appear to have distinct spatiotemporal dynamics compared to other mitochondrial contacts in terms of formation, transport, and structures formed (Huang et al., 2013). The role of mitochondria nanotunnels in cardiac pathologies remain to be fully characterized.

Mitochondria in adult cardiomyocytes are divided into three groups based on their location and function: interfibrillar, subsarcolemmal, and perinuclear (Palmer et al., 1977). Interfibrillar mitochondria comprise most of the mitochondrial population and are found between myofibrils. They have tubular cristae that are involved in ATP production for cardiomyocyte contraction and Ca^2+^ signaling. Subsarcolemmal mitochondria are located under the sarcolemmal membrane and have lamellar cristae used for energy generation for ion channels and cell signaling (Riva et al., 2005; Ong et al., 2017). Perinuclear mitochondria are clustered around the sides of the nucleus and are postulated to aid in transcriptional regulation (Santel and Fuller, 2001; Ong et al., 2017). These different types of mitochondria are all functionally linked, thus participating in electrical conduction coupling among cardiac cells (Amchenkova et al., 1988).

**Figure 1:**
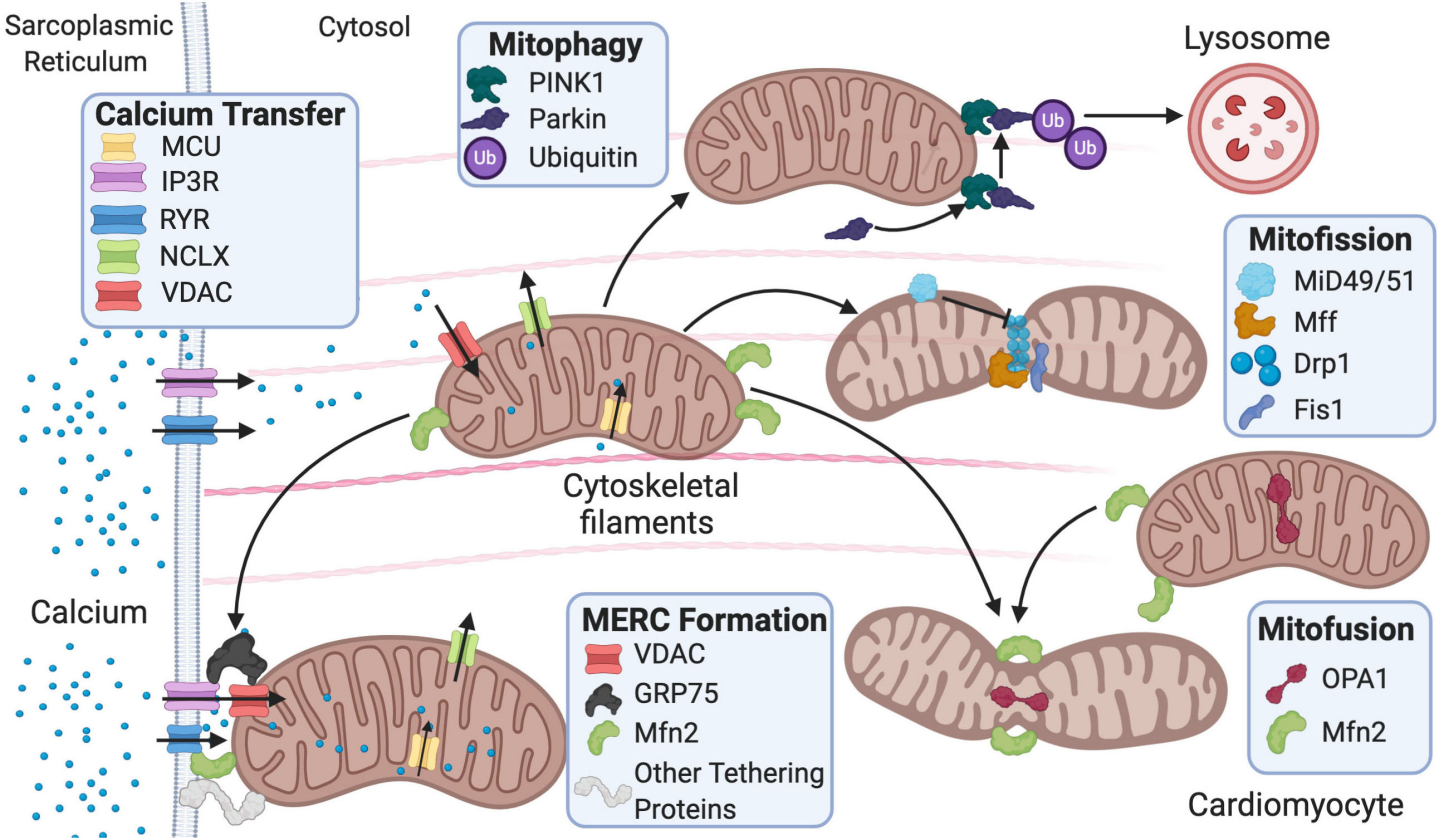
Fusion and fission protein dynamics and how sarcoplasmic reticulum (SR) and mitochondrial communication participate in these processes. Fusion and fission are both regulated by GTPases. Mitofusion occurs through tethering of both mitochondrial membranes and then fusion via Mfn1/2 and OPA1. Mitofission occurs via Drp1-mediated constriction of the mitochondria, which is inhibited by MiD49/51. Fis1 acts as a recruiting molecule for Drp1, while Mff is the receptor for Drp1. MERC formation is mainly regulated by Mfn2 but also involves the IP_3_R-GRP75–VDAC complex and other numerous proteins. Mitophagy is initiated by PINK1 phosphorylation of Parkin, leading to targeting of the mitochondria for degradation by the lysosome. MERC, mitochondrial-ER contact; IP_3_R, inositol 1,4,5-trisphosphate receptor; GRP75, glucose-related protein 75; VDAC, voltage-dependent anion channel; RyR, ryanodine receptor; NCLX, Na^+^/Ca^2+^ exchanger, Mfn, mitofusin; Opa1, optic atrophy 1; Fis1, mitochondrial fission 1 protein; Drp1, dynamin-related protein 1 Mff, mitochondrial fission factor; MiD49/51, mitochondrial dynamic proteins 49/51; PINK1, PTEN-induced kinase 1.

Mitochondrial ultrastructure and dynamics are integral for cardiac tissue maintenance and homeostasis. Cardiac pathophysiological conditions have been linked to alterations in these processes and include swelling, loss or reorientation of cristae, distortion of configuration, or vacuoles found in the inner or outer compartments (Hoppel et al., 2009). Of note, cardiac pathologies have been linked to the presence of giant mitochondria. The clearance of dysfunctional mitochondria is a significant component in mitochondrial network health and involves a process known as mitophagy (Galluzzi et al., 2017). Giant mitochondria can be formed by either fusion of two mitochondria via increased expression of mitofusins (Mfn) or by the growth of a single mitochondrion (Kraus and Cain, 1980; Arbustini et al., 1998; Santel and Fuller, 2001). However, the mechanism of how giant mitochondria directly participate in cardiac dysfunction remains largely understudied. Mitochondrial morphology is regulated by fission and fusion (Figure 1), a balanced process required for a healthy cell.

Mitochondrial fission is used in repair, cell division, and mitophagy, while fusion is used to elongate and exchange matrix components. Dysregulation of these processes is observed in various cardiovascular pathologies and linked to disease progression (Vásquez-Trincado et al., 2016). Fission is regulated by GTPases mitochondrial fission 1 protein (Fis1), dynamin-related protein 1 (Drp1), mitochondrial fission factor (Mff), and mitochondrial dynamic proteins of 49 and 51 kDa (MiD49/51). These outer mitochondrial membrane proteins act as receptors for Drp1 and work to recruit Drp1 to the mitochondria as Drp1 lacks a mitochondrial targeting sequence (Gandre-Babbe and Van Der Bliek, 2008; Losón et al., 2013). They are potentially redundant as each receptor can interact with Drp1 independently; nevertheless, it remains unclear whether these receptors compete or work in consortium in the overall process (Yu et al., 2020). It is reported that in 293T cells, MiD49/51 and Mff may compete with each other to interact with Drp1 (Yu et al., 2017). The importance of Fis1 in translocating Drp1 to the mitochondria remains controversial. Studies have reported that knockout of human Fis1 (hFis1) in HeLa and HCT116 cells does not affect Drp1 binding to the mitochondria (Lee et al., 2004; Otera et al., 2010), while another study using mouse embryonic stem cells reported that Fis1 is heavily involved in recruiting Drp1 to the mitochondria—potentially more than Mff (Seo et al., 2020).

The importance of mitochondrial fission has been highlighted by studying how the downregulation of Drp1 causes mitochondrial elongation, hindrance of mitophagy, increased probability for mitochondrial permeability transition pore (mPTP) opening, and altered stress responses. Genetic mouse models of Drp1 downregulation developed cardiac dysfunction and possessed increased susceptibility for ischemia/reperfusion (I/R) injury (Ikeda et al., 2015). Moreover, mitochondrial fission helps cell repair by extruding damaged components, which is then targeted for removal and degradation via mitophagy. Drp-1-mediated fission has been shown to act as an adaptive mechanism during cellular stress, although its role in exercise is debatable. One study found Drp1-mediated fission in mice to amplify cardiac and mitochondrial function during exercise (Coronado et al., 2018). A more recent study also performed in mice found fission mediated by Mcl-1—a potential receptor for Drp1—to reduce nutrient deprivation-induced cell death but, on the other hand, reduce mitochondrial ability to adapt to a sudden increase in energy demand and workload (Moyzis et al., 2020). As a balanced regulation of fission is necessary for healthy cells, overexpression of Drp1 also induced mitochondrial dysfunction and excessive cell death in the cardiomyocytes via apoptosis (Ikeda et al., 2015).

Drp1 is extensively regulated by many posttranslational mechanisms, including phosphorylation of residues Ser-637 and Ser-616. Phosphorylation of Ser-616 activates Drp1, promoting cytosolic to mitochondrial translocation, and inducing fission (Marsboom et al., 2012); on the contrary, phosphorylation of Ser-637 retains Drp1 in the cytoplasm (Chang and Blackstone, 2007). Studies have reported that Drp1 is phosphorylated at Ser-637 and dephosphorylated at Ser-616 in starvation conditions (Rambold et al., 2011), while the opposite is true during hyperglycemic conditions (Gawlowski et al., 2012). The inhibition of Drp1 during starvation would lead to fusion and increase mitochondrial respiration (Rambold et al., 2011), supporting the notion that mitochondrial dynamics are linked to the metabolic state of cells (Seo et al., 2020).

Fusion is regulated by GTPases mitofusin 1/2 (MFN1/2) and optic atrophy 1 (OPA1) (Figure 1). Fusion occurs stepwise with mitochondria tethering together first via MFN1/2. Then, the outer mitochondrial membranes fuse as a result of MFN activity (Chen and Chan, 2005). Last, the inner mitochondrial membranes are fused via OPA1 (Cipolat et al., 2004). Studies have reported that MFN1/2 can act redundantly during fusion, and ablation of both results in mitochondrial structural abnormalities and dilated cardiomyopathy in the postnatal heart (Papanicolaou et al., 2012). Postnatally, the heart undergoes rapid proliferation of mitochondria and an increase in respiratory function. Thus, the ablation of both MFNs is lethal in murine models (Papanicolaou et al., 2012) as is the case with homozygous OPA1 mutants (Piquereau et al., 2012). Interestingly, no alteration in mitochondrial respiration was found in mice with a heterozygous OPA1 deletion, but there was mitochondrial network remodeling and increased sensitivity to hemodynamic stress (Piquereau et al., 2012). Ubiquitination of MFNs promotes degradation, which allows for unopposed mitochondrial fission during the removal of damaged mitochondria via mitophagy (Gegg et al., 2010). For mitophagy to occur, mitochondria need to undergo fission, thus inhibition of fusion aids this process. Drp1, on the other hand, was not found to be ubiquitinated during mitophagy as expected (Gegg et al., 2010). The homeostatic balance between fusion and fission is vital for the maintenance of healthy mitochondria in the heart. Similar to “yin and yang,” these opposite and contrary forces are complementary and interconnected, such that dysregulation in either of these processes leads to mitochondrial dysfunction related to cardiac pathologies.

Interestingly, in addition to ER connections, the role of mitochondria has extended to other subcellular membranes. Recent studies have shown that mitochondria can shed mitochondrial-derived vesicles (MDVs) with specific cargoes to the peroxisome or the late-endosome (Neuspiel et al., 2008; Braschi et al., 2010; Soubannier et al., 2012). While these MDVs are distinct from mitophagy-related mechanisms, MDVs have been linked to alterations in cellular physiology and metabolism, although precise mechanisms of action remain largely unknown requiring further investigation.

## Calcium Signaling, Apoptosis, and Ferroptosis

Calcium entry in the mitochondria plays an important role in metabolic regulation and cell death mechanisms. A prominent role for mitochondrial calcium entry has been attributed to the mitochondrial calcium uniporter (MCU) complex. Surprisingly, global ablation of MCU has no overt baseline phenotype as these animals had an apparent normal lifespan. While acute mitochondrial calcium entry in the MCU knockout was absent, mitochondrial calcium levels were reduced but existent in mitochondria derived from MCU knockout mice, suggesting the existence of other forms of mitochondrial calcium entry (Pan et al., 2013). Although perhaps moderately speculative, this could be partially attributed to connexin 43 (Cx43), as calcium is permissive through these channels, and Cx43 has been reported to be localized in the mitochondria (Gadicherla et al., 2017). Moreover, the viability of MCU knockout mice depended on the genetic background, as MCU KO was embryonically lethal in C57/BL6 but not in CD1 background (Murphy et al., 2014). Interestingly, cardiac-specific ablation of MCU led to cardioprotection post-I/R (Luongo et al., 2015). Additionally, a variety of proteins have been reported to regulate the MCU complex, among which are spastic paraplegia 7 (SPG7), mitochondrial calcium uptake 1 (MICU1), mitochondrial calcium uniporter 1 (MCUR1), and essential MCU regulator (EMRE) (Mallilankaraman et al., 2012a,b; Shanmughapriya et al., 2015; Vais et al., 2016). Studies over decades have shown that mitochondrial calcium overload can lead to the opening of the mitochondrial permeability transition pore (mPTP) and subsequent cell death. Although mPTP pore function is widely accepted to be vital for cell death mechanisms, the molecular identity of the pore-forming channel remain largely debated. Novel evidence has pointed to an additional function for the F-ATPase, particularly for the c-subunit of the ATPase as the pore-forming channel (Alavian et al., 2015; Urbani et al., 2019). This knowledge has been counter argued by John Walker's group (Nobel Laureate 1997), where persistent permeability transition pore is observed in human mitochondria devoid of assembled ATP synthase (Carroll et al., 2019). For a more extensive review of mitochondrial calcium and its regulators, we defer to another recent review (Garbincius et al., 2020).

Ca^2+^ signaling can occur via a physical connection between the mitochondria and ER, which is thought to be mediated by MFN2 tethering of both organelles (Figure 1) (De Brito and Scorrano, 2008). Nevertheless, there are several controversies surrounding the functional role of MFN2. For instance, reports have shown opposing effects of Mfn2 deficiency in the susceptibility to I/R injury. One study found decreased susceptibility to I/R injury due to delayed mPTP opening from decreased Ca^2+^ intake (Papanicolaou et al., 2011). Another study found increased susceptibility to I/R injury as a result of dysfunctional autophagy and disturbed mitochondrial ultrastructure (Zhao et al., 2012). It is thought that this discrepancy may be due to differences in timing and location as both studies used murine models (Zhao et al., 2012). Moreover, there have been opposing reports on the impact of ablation of Mfn2 on mitochondria–ER junctional distance. One study found the distance between the two organelles to increase by approximately 30% (Chen et al., 2012), while another found no difference in distance (Papanicolaou et al., 2012). It is possible that the discrepancy is due to timing of the Mfn2 gene deletion as the former study ablated Mfn2 during embryonic stages in mice, and the latter ablated Mfn2 after birth in mice (De la Fuente and Sheu, 2019). This is intriguing as it remains unclear how the mitochondria respond to rapid cytosolic Ca^2+^ transients from the ER in contracting cardiac myocytes (Andrienko et al., 2009). Two models describe how cardiac mitochondria interpret Ca^2+^ transients released by the sarcoplasmic reticulum (SR). One model states that mitochondria imports and releases Ca^2+^ with each heartbeat, while the other proposes that mitochondria gradually take up Ca^2+^ until a steady-state environment is reached (Sedova et al., 2006). It is hypothesized that the former model is used in larger animal models relative to mice, while the latter is found mainly in smaller animal models such as mice (Griffiths, 1999). Studies have shown that in addition to Ca^2+^ cycling, cardiac mitochondria are also involved in cellular Ca^2+^ buffering. It was found that in neonatal cardiomyocytes, Ca^2+^ is taken up by the mitochondria in systole and released back into the cytosol during diastole. Between 1% and 15% of cytosolic Ca^2+^ is thought to be buffered by cardiac mitochondria (Drago et al., 2012). Mitochondrial fusion frequency is also coupled with the frequency of Ca^2+^ transient oscillations, rather than sustained transients, and cardiomyocyte contractions (Eisner et al., 2017). In cases where a ryanodine receptor (RyR2) channel mutation is present in murine models, pathologies arise due to altered Ca^2+^ oscillations and inability of cardiomyocytes to contract (Lavorato et al., 2017) linked to fewer fusion events (Eisner et al., 2017). Further studies are needed to expand our understanding on the role of MFN2, ER–mitochondrial Ca^2+^ communication, and susceptibility to cardiac injury.

Mitochondrial Ca^2+^ is an important regulator of cell death. In fact, apoptosis is a major form of cell death in cardiomyocytes that occurs in response to ischemia or metabolic stress linked to decreased ATP levels, dysfunctional electron transport chain (ETC), or excessive oxidative stress. It involves mitochondrial fragmentation, increased mitochondrial permeability caused by Ca^2+^ influx, proapoptotic protein assembly, and cytochrome C release to the cytosol. Pro-apoptotic factors BCL2-interacting protein 3 (BNip3) and BNIP3-like (Nix) form heterodimers with antiapoptotic factors such as B-cell lymphoma—extra large (BclxL) and B-cell lymphoma 2 (Bcl2), which then permit activation of proapoptotic proteins BCL2-associated X (BAX) and BCL2 antagonist killer (BAK). BAX and BAK are pore-forming proteins and increase mitochondrial outer membrane permeability, allowing for the release of cytochrome c into the cytosol and initiation of apoptosis (Gálvez et al., 2006; Dorn, 2010). Apoptosis signaling can be initiated through a secondary pathway when Fis1 on the mitochondrial outer membrane binds to BAP31 on the ER membrane (Iwasawa et al., 2011). Drp1 is not only involved in mitochondrial fission, as it can also promote mitochondrial fragmentation and subsequent apoptotic signaling (Frank et al., 2001). Mitochondrial fragmentation is a component of apoptosis that occurs through mitochondrial fission (Oettinghaus et al., 2016). Nevertheless, Drp1 is not essential for cell death as other proapoptotic proteins can be released in the cytosol to induce apoptosis in the absence of Drp1. Contrarily, Drp1 may induce apoptosis on its own without other proapoptotic proteins (Oettinghaus et al., 2016).

The Bcl-2 family of proteins are paramount regulators of cellular apoptosis. Evidence suggests that these proteins work by decreasing Ca^2+^ release from the ER, increasing IP_3_R channel leakage, dampening Ca^2+^ overload in the mitochondria, and mPTP opening probability in response to stress (Bittremieux et al., 2016). mPTP opening dissipates mitochondrial membrane potential, causing swelling and release of proapoptotic factors into the cytoplasm (De Giorgi et al., 2002). Thus, increased mitochondrial Ca^2+^ influx promotes mPTP opening, followed by ATP depletion and cell death (Dorn, 2010).

Ferroptosis is a newly defined form of apoptosis that is dependent on iron. It results from the accumulation of lipid ROS driven by the deficiency of the scavenging antioxidant, glutathione. Iron is used as a catalyst in lipid peroxide-generating reactions; thus, deficiency of ferritin or iron concentration will drive ROS production. Ferroptosis is associated with changes in mitochondrial morphology, such as cristae enlargement and mitochondrial fragmentation. A study using human fibrosarcoma HT1080 cells and mouse embryonic fibroblasts showed that mitochondrial tricarboxylic acid (TCA) cycle and ETC function are essential for potent ferroptosis (Gao et al., 2019). The TCA cycle is required as glutamine, and downstream TCA cycle metabolites are used in the initiation of ferroptosis, while the ETC is necessary to ensure ROS production. Studies were performed in HT1080 cells depleted of mitochondria via Parkin-mediated mitophagy, which caused them to become resistant to ferroptosis (Gao et al., 2019).

Inhibition of ferroptosis has been reported to reduce I/R-related cardiac pathology, protect from cardiomyopathy, and reduce HF incidence (Fang et al., 2019). Heme oxygenase-1 (Hmox1) catalyzes heme degradation, which releases free iron, leading to ferroptosis and HF. Hmox1 is considered to play paradoxical roles on its status as a cardioprotective protein. One study found that Hmox1 can serve a cardioprotective role as overexpression in mice can protect against I/R injury and permanent coronary ligation-induced HF (Wang et al., 2010). On the other hand, another study found that inhibition of Hmox1 in mice is cardioprotective, in a manner that is similar to the effects of iron chelation (Fang et al., 2019). Similarly, a third study showed that overexpression of Hmox1 in murine models leads to spontaneous HF by 1 year of age despite being protected against isoproterenol-induced cardiomyopathy (Allwood et al., 2014). Further studies are required to delineate precise mechanisms of Hmox1 and iron-related cardiac pathologies.

## Membrane Contacts and Communication Between Endoplasmic Reticulum and Mitochondria

The mitochondrion has numerous contacts with other organelles such as peroxisomes and the plasma membrane, but the most well-characterized interactions involve ER membranes—an organelle involved in lipid and protein synthesis (Szymański et al., 2017). The peroxisome is an organelle used for oxidation and lipid synthesis, and the plasma membrane is a semipermeable membrane that surrounds the cell. The ultrastructural organization of the contact sites between the mitochondria and ER is termed the mitochondrial–ER contact (MERC), while the collection of proteins and lipids that form the MERC is called the mitochondrial-associated ER membrane (MAM) (Figure 1) (Giacomello and Pellegrini, 2016). An early study using HeLa cells showed that up to 20% of the mitochondrial surface is near ER membranes (Rizzuto et al., 1998). Later on, another study using RBL-2H3 and H9c2 cells proposed that all mitochondria are in contact with the ER (Csordás et al., 2010). While the percentage of ER-mitochondrial membrane contacts remains debatable, it is well-established that the outer membrane of the mitochondria, MAMs, and ER membranes are well-connected, and even the inner membrane of the mitochondria continues to influence pattern, structure, and physiology of adjacent mitochondria (Vincent et al., 2017). Specific proteins have been reported to play a significant role in tethering the ER and mitochondria (Lee and Min, 2018). While some proteins are common to various cellular membranes, others are specific to MAMs.

Although the MERC tethering system has been extensively investigated in yeast and mammalian cells, the exact composition and potential interactions have not been fully characterized (Lee and Min, 2018). In yeast cells, a tethering complex called the ER–mitochondria encounter structure (ERMES) connects the ER to the mitochondrial outer membrane. It is comprised of maintenance of mitochondrial morphology protein 1 (MMM1), mitochondrial disruption and morphology protein 10 (MDM10), mitochondrial disruption and morphology protein 12 (MDM12), and mitochondrial disruption and morphology protein 34 (MDM34) (Kornmann et al., 2009). Specific protein localization via confocal fluorescence microscopy was determined in wild-type and mutant cells that expressed each protein independently. MMM1 was found to be on the ER membrane, MDM10/34 was observed on the mitochondrial outer membrane, and MDM12 was found to be the linker molecule between the two subcellular components (Kornmann et al., 2009).

Mammalian cells lack an ERMES counterpart and are thought to have a more complicated protein interface than yeast cells (Lee and Min, 2018). In mammals, MFN2 is a tethering protein (De Brito and Scorrano, 2008), independent of its role in mitochondrial fusion. Although it is unclear whether MFN2 increases the mito–ER junctional distance, as stated before, cardiac-specific MFN2 knockout studies have led to decreased mitochondrial sensitivity to the SR Ca^2+^ release (Chen et al., 2012; Papanicolaou et al., 2012). The amount of SR-associated RyRs in cardiomyocyte MAMs without MFN2 was significantly lower than wild-type cells despite the overall unchanged cardiac content of RyRs. These effects are either less pronounced or not observed in cells where MFN1 is knocked out (Chen et al., 2012). Nonetheless, in cardiac cells with dual MFN1 and MFN2 ablation, mitochondrial fragmentation, and rapidly lethal dilated cardiomyopathy was observed (Chen et al., 2012).

Other major proteins involved in tethering include vesicle-associated membrane protein-associated protein-B (VAPB), protein tyrosine phosphatase-interacting protein-51 (PTPIP51), glucose-related protein 75 (GRP75), inositol 1,4,5-trisphosphate receptor (IP_3_R), voltage-dependent anion channel (VDAC), B-cell receptor-associated protein 31 (BAP31), FIS1, and PDZ domain-containing 8 (PDZD8) (Lee and Min, 2018). In addition to tethering, these proteins play a part in regulating Ca^2+^ buffering, lipid processing, mitochondrial fusion, and autophagy. The abundance, localization, and/or interactions of these proteins often lead to different outcomes, further masking our understanding of this complex process (Lee and Min, 2018). For instance, VAPB is an ER membrane protein, while PTPIP51 is an outer mitochondrial membrane protein. Together, they participate in Ca^2+^ regulation and autophagy. Overexpression of both proteins impairs autophagy and increases mitochondrial Ca^2+^ uptake, while deficiency of both has the opposite effect (Gomez-Suaga et al., 2017). IP_3_Rs are located on the ER membrane and facilitate Ca^2+^ efflux toward the mitochondria, while VDAC is situated on the outer mitochondrial membrane and allows for Ca^2+^ influx into the mitochondria. GRP75 is a cytosolic linker that bridges IP_3_R and VDAC and promotes delivery of Ca^2+^ into the mitochondria. Cardiac mitochondrial Ca^2+^ uptake is then mediated via the MCU complex in conjunction with VDAC (Szabadkai et al., 2006; Xu et al., 2018). A study analyzing the downregulation of GRP75 in neuronal cells showed reduced ER–mitochondrial coupling and attenuation of Ca^2+^ transfer from the ER to the mitochondria (Honrath et al., 2017). This allowed for mitochondrial resistance to Ca^2+^ overload during oxidative stress. Nevertheless, it remains unclear whether GRP75 inhibition holds potential as a pharmacological target against oxidative stress in cardiomyocytes (Honrath et al., 2017). BAP31 is located on the ER membrane, and FIS1 can be found on the mitochondrial outer membrane. Together, they regulate a secondary pathway for initiation of apoptosis. FIS1 elicits apoptosis signaling via an early and specific caspase-like protease activity that facilitates the cleavage of BAP31 into its proapoptotic form (Iwasawa et al., 2011). This cleavage allows for the recruitment and cleavage of procaspase-8 into caspase-8, a powerful activator of apoptosis. The BAP31–FIS1 complex releases Ca^2+^ downstream from the ER that further amplifies cell death via a positive feedback loop (Iwasawa et al., 2011). PDZD8 is another crucial tethering protein that participates in Ca^2+^ dynamics between the ER and mitochondria. PDZD8 knockdown in neuronal cells significantly decreased mitochondrial Ca^2+^ import despite no change in the Ca^2+^ import machinery, thus implicating the protein in ER–mitochondrial tethering (Hirabayashi et al., 2017). In this latter study, PDZD8 was identified as an ortholog to MMM1 in the yeast ERMES complex, although another study using phylogenetic analyses showed it to be a paralog instead (Wideman et al., 2018).

The ER–mitochondrial tether additionally plays a role in lipid trafficking (Rusiñol et al., 1994). Specific proteins and enzymes involved in lipid synthesis and transfer are enriched in the MAM subdomain of the ER. Examples of these proteins include acyl-coenzyme A cholesterol acyltransferase (ACAT1), phosphatidylserine (PS) synthase, phosphatidylethanolamine N-methyltransferase 2 (PEMT2), fatty acid coenzyme A (CoA) ligase 4 (ACS4), and diacylglycerol O-acyltransferase 2 (DGAT2) (Szymański et al., 2017). ACS4 is used to synthesize acyl CoA used as a precursor for triacylglycerols (TAGs), and DGAT2 catalyzes the final step in TAG synthesis. In a study that examined DGAT2, a known mitochondrial targeting sequence was found in the N-terminus (Stone et al., 2009). MAMs are crucial for the movement of phospholipids between the ER and mitochondria (Hernández-Alvarez et al., 2019). A study using human models of non-alcoholic steatohepatitis and mouse models of non-alcoholic liver disease found decreased levels of Mfn2. In the same study, liver-specific Mfn2 knockout in mouse models resulted in disturbances of ER–mitochondrial PS transfer, revealing a novel mechanism in the development of liver disease (Hernández-Alvarez et al., 2019). Mitochondria import PS synthesized in the ER via PS synthase, which can be decarboxylated into phosphatidylethanolamine (PE). Synthesized PE is able to be exported back to the ER, where it can be methylated via PEMT2 into phosphatidylcholine (PC) (Ridgway and Vance, 1987). ACAT1 catalyzes the synthesis of cholesterol esters, which allows for control of the equilibrium between cytosolic and membrane-bound cholesterol (Puglielli et al., 2001). Cholesterol transfer to the mitochondria from the ER provides material for steroid synthesis. Cholesterol transfer, along with membrane organization and stability, is regulated by caveolin 1 (CAV1), which is abundantly present in MERCs, although its role is controversial. One study using a genetic knockout model found CAV1 to be essential for MERC recruitment and regulation (Sala-Vila et al., 2016), while another study using an inducible CAV1 expression system found CAV1 to cause impairment of MERC communication and remodeling (Bravo-Sagua et al., 2019).

Mitochondrial dynamics are also influenced by MERC tethering, where phospholipid composition of membranes regulates mitochondrial fusion and fission. The main two phospholipids involved are phosphatidic acid (PA) and cardiolipin (CL) (Yu et al., 2020). The *de novo* mitochondrial synthesis of CL is followed by cycles of CL deacylation and reacylation, which can result in the generation of an array of CL species. Increased exposure of CLs occurs on the outer mitochondrial membrane during mitochondrial stress, where they can serve as binding sites for signaling proteins (Schlame and Haldar, 1993; Osman et al., 2011). CL works in the outer mitochondrial membrane to stimulate pro-fission GTPase Drp1, while PA interacts with Drp1 to inhibit it and promote fusion (Bustillo-Zabalbeitia et al., 2014; Adachi et al., 2016). CL can also promote fusion by interacting with OPA1 on the inner membrane, while PA can stimulate fusion by participating in it with MFN (Choi et al., 2006; Ban et al., 2017).

## Role of Mitochondria in Cardiac Health

Mitochondria are the main energy source in cells and are especially important in energy-intensive organs such as the heart. Energy is produced in the form of ATP via oxidative phosphorylation. A balance in the concentration of ATP, adenosine diphosphate (ADP), creatine phosphate (CrP), and inorganic phosphate (Pi) are essential for a healthy heart. In the heart, changes in ATP, ADP, and CrP levels are detected to ensure that transient changes in their levels can cause large heart rate alterations (Balaban et al., 1986). The levels of ATP are thought to be low due to fast turnover and intensive energetic demand (Chistiakov et al., 2018). The enzyme creatine kinase produces phosphocreatine, which is involved in ATP buffering in the myocardium. When there are conditions of high ATP turnover, phosphocreatine concentration is altered to increase oxidative phosphorylation (Bark, 1980). Creatine kinase exists in an octameric form, which is very reactive, and a dimeric form, which is slower. In heart disease, creatine kinase is found predominantly in the slow dimeric form due to disassociation of the octamer. This causes dysfunctional oxidative phosphorylation and less ATP production due to impaired phosphocreatine hydrolysis (Soboll et al., 1999).

The heart obtains over half of its energy from fatty acid oxidation, though it can preferentially utilize glucose instead of lipids depending on energy demands (Ljubkovic et al., 2019). The level of malonyl CoA determines this switch, as increased malonyl CoA levels are associated with increased glucose oxidation and decreased fatty acid oxidation. In the heart, high activity of malonyl CoA decarboxylase (MCD), which converts malonyl CoA to acetyl CoA, maintains cardiac fatty acid substrate preference (Dyck et al., 2004). Studies have proposed that a switch in substrate availability can be altered by insulin signaling (Ljubkovic et al., 2019). In pathologic conditions such as HF or dilated cardiomyopathy, cardiac metabolism shifts toward glucose oxidation under resting conditions. However, under stress, this switch becomes detrimental due to disrupted insulin signaling and inefficient glucose uptake (Neglia et al., 2007).

An efficient mitochondrial ETC uses 98% of the electrons for ATP production (Chistiakov et al., 2018). The small percentage of electrons that escape the ETC and generate superoxide radicals are normally quickly broken down by superoxide dismutase (Boveris et al., 1976). Although the exact mechanism is not fully characterized, a small amount of ROS production can be cardioprotective by triggering protective mechanisms before and after I/R injury (Murry et al., 1986; Zhao et al., 2003). These processes are referred to as preconditioning and postconditioning, respectively. Uncoupling of the ETC results in ROS overproduction and inefficient ATP synthesis. In addition to cellular death, excessive ROS generation can induce atherogenesis through increasing vessel inflammation, oxidized low-density lipoprotein attachment to the vessel wall, endothelial dysfunction, and plaque-induced rupturing (Förstermann et al., 2017).

**Figure 2:**
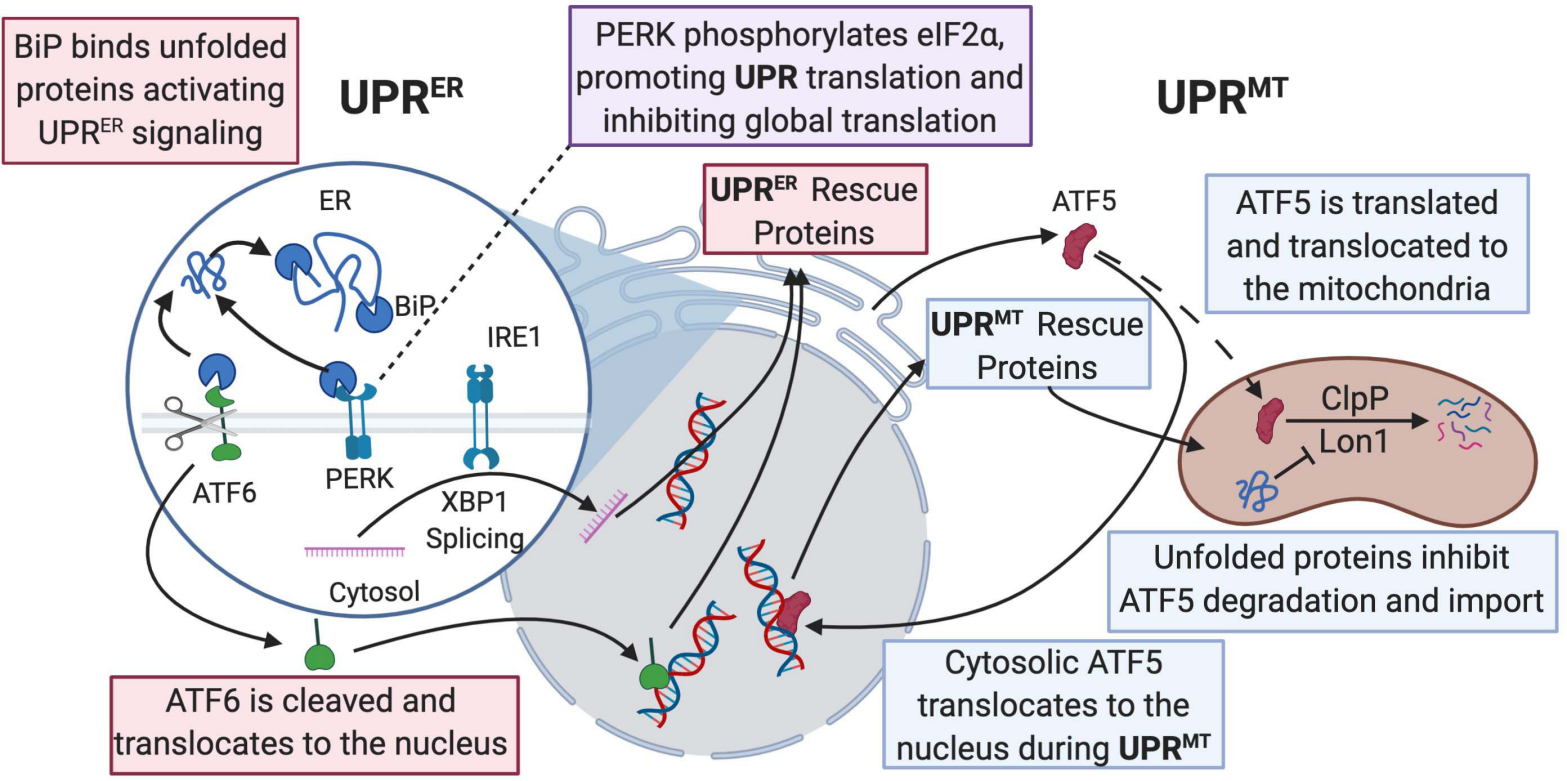
UPR^ER^ and UPR^mt^ mechanistic commonalities and differences are described in this figure. BiP binds to misfolded proteins and promotes UPR^ER^ signaling, allowing for activation of the ATF6, PERK, and IRE1 pathways. ATF6 acts as a transcription factor and activates production of protein-folding machinery. PERK phosphorylates eIF2α, promoting translation of both UPR^ER^ and UPR^mt^ protein-folding machinery and downregulating global translation. IRE1 splices Xbp1 and allows the spliced product to transcribe protein chaperones in the nucleus. In the ATF5 pathway of UPR^mt^, ClpP, and Lon1 diverge from degradation of ATF5 to degradation of misfolded proteins and the accumulation of ATF5 results in transcription of UPR-associated proteins in the nucleus. Dashed line is ATF5 movement in normal physiological conditions; solid line is enhanced when UPRmt is activated. UPR, unfolded protein response; BiP, binding immunoglobulin protein; ATF, activating transcription factor; PERK, protein kinase RNA-like endoplasmic reticulum kinase; ClpP, ATP-dependent Clp protease proteolytic subunit; Lon1, Lon protease homolog 1; IRE1, inositol-requiring kinase 1; Xbp1, X-box binding protein 1; eIF2α; eukaryotic translation initiation factor 2A.

A healthy heart depends on balanced maintenance of contractile function and constant energy production. Regulation of cellular protein integrity in the heart is termed proteostasis, which involves heat shock response chaperones, autophagy, the ubiquitin–proteasome system, and the unfolded protein response (UPR) (Figure 2) (Arrieta et al., 2020). These processes all act when the mitochondria are under cellular stress. The mitochondrial protein folding environment is complex and the mitochondrial UPR (UPR^mt^) is not as well-researched as its counterpart, the ER UPR (UPR^ER^) (Li et al., 2019). UPR^ER^ will be discussed in the next section.

At normal physiological conditions, when UPR^mt^ is not activated, mitochondrial precursors must be transcribed and translated in the cytoplasm where they are maintained in an unfolded state guided by cytosolic heat shock protein (HSP) chaperones prior to being imported into the mitochondrion, where proteins are then folded (Priesnitz and Becker, 2018). Import stress such as reduced efficiency due to mitochondrial membrane depolarization or mismatch in production of respiratory complex subunits from mtDNA vs. nuclear DNA, leads to the buildup of misfolded or unfolded proteins (Figure 2) (Quirós et al., 2015; Rolland et al., 2019). These unfolded proteins activate the UPR^mt^, resulting in the transcription and translation of HSP10/60, and proteases, ATP-dependent Clp protease proteolytic subunit (ClpP) and lon protease homolog 1 (Lon1), to fold the proteins or repair damaged proteins (Martinus et al., 1996; Zhao et al., 2002; Haynes et al., 2007). The initiation of the UPR^mt^ can occur through two separate pathways. One pathway involves activating transcription factor 5 (ATF5), a transcription factor imported into the mitochondria, where Lon1 and ClpP degrade ATF5 under normal conditions. However, under cellular stress, the accumulation of misfolded proteins in the mitochondria prevents mitochondrial import of ATF5. Decreased mitochondrial import of ATF results in increased Lon1- and ClpP-induced degradation of the misfolded proteins in place of ATF5. The reduced mitochondrial import of ATF5 leads to increased trafficking of ATF5 to the nucleus, resulting in the transcription of UPR-associated proteins, promoting cellular defenses against stress signaling (Nargund et al., 2012; Fiorese et al., 2016). In addition to directly influencing protein translation, Lon1 has been shown to influence transcription indirectly. During activation of UPR^mt^, Lon1 degradation of mitochondrial ribonuclease P catalytic subunit (MRPP3) increases MRPP3 turnover, leading to an accumulation of Mrpp3 RNA precursors resulting in impairment of mitochondrial translation (Münch and Harper, 2016). Knockdown of ClpP caused alteration of mitochondrial morphology, excessive ROS production, and a breakdown of oxidative phosphorylation even under non-stressed conditions (Deepa et al., 2016). This study suggested a more global role of ClpP for mitochondrial maintenance than impacting the UPR^mt^. The second pathway is initiated by protein kinase RNA-like endoplasmic reticulum kinase (PERK) phosphorylating eukaryotic translation initiation factor 2A (eIF2α), which inhibits protein translation and activates ATF4/5 and C/EBP homologous protein (CHOP) translation. C-Jun N-terminal kinase 2 (JNK2) also binds to the transcription factor C-Jun, which results in the transcription of CHOP. ATF4/5 and CHOP all assist in transcribing other UPR^mt^ proteins (Aldridge et al., 2007; Baker et al., 2012; Verfaillie et al., 2012). The cumulative effects of both UPR^mt^ pathways result in reduced overall mitochondrial protein translation and increased translation of specific proteins that assist with refolding or degrading unfolded or misfolded proteins.

Mitophagy is another process that plays a role in proteostasis. Unlike the UPR^mt^, which occurs when mitochondria are still salvageable, mitophagy occurs when mitochondria are irreparably damaged (Pickles et al., 2018). Mitophagy is initiated by phosphorylation and mitochondrial recruitment of ubiquitin ligase Parkin by PTEN-induced kinase 1 (PINK1) during stress (Kane et al., 2014). Phosphorylated Parkin recruits autophagy receptors by placing ubiquitin chains on mitochondrial outer membrane proteins (Lazarou et al., 2015). p53-induced inhibition of Parkin exacerbated cardiac aging and dysfunction, suggesting that activation of mitophagy could be a potential target for therapy in diseases related to aging or mitochondrial dysfunction (Hoshino et al., 2013; Pires Da Silva et al., 2020). Interestingly, Drp1—a key protein involved in mitochondrial fission—is also essential for mitophagy. Deficiency of Drp1 resulted in loss of mitophagy, promotion of cardiac dysfunction, and increased susceptibility to I/R injury (Ikeda et al., 2015). Mitophagy is a particularly fascinating topic of research as it is a reparative protective mechanism that may become pathological when uncontrolled.

Alterations in epigenetic regulation are known to promote various pathologies. Recent studies have shown the importance of metabolite diversion from bioenergetic pathways to nuclear epigenetic regulation. Mitochondrial calcium exchange has been shown to participate in myofibroblast activation by promoting shuttling of α-ketoglutarate from the mitochondria to the nucleus where it activates histone demethylases enhancing chromatin accessibility (Lombardi et al., 2019). The concept of retrograde signaling is strongly supported by oncometabolites such as 2-hydroxyglutarate, which are enhanced in gain-of-function mutations in isocitrate dehydrogenases, shown to participate in epigenetic modification of various tumors (Shim et al., 2014). How mitochondrial dynamics may alter retrograde signaling and/or be involved in cellular differentiation as it pertains to cardiac pathologies remain to be fully characterized.

## Role of Endoplasmic Reticulum (Sarcoplasmic Reticulum) in Cardiac Health

The ER is the bridge between many different types of membranes and organelles. Important membrane junctions between the ER and other organelles include the ER–endosome (Friedman et al., 2013), ER–peroxisome (Knoblach et al., 2013), ER–Golgi (Peretti et al., 2008), and ER–mitochondria junctions (Shore and Tata, 1977). Along with its extensive contacts, the ER continuity allows other organelles to communicate rapidly via the ER network (Wozniak et al., 2009). A specialized form of the ER exists in cardiomyocytes called the sarcoplasmic reticulum (SR) which acts as a reservoir for Ca^2+^ to facilitate intracellular Ca^2+^ release and storage. Upon depolarization, Ca^2+^ enters the cell and binds to RyRs, which induces calcium-induced Ca^2+^ release (CICR) (Otsu et al., 1990). CICR in the SR is crucial in muscle contraction and relaxation (Terracciano and MacLeod, 1997). Other proteins found in the SR involved in Ca^2+^ signaling include Ca^2+^-ATPases (SERCAs), which work to import Ca^2+^ into the ER and IP_3_Rs, which release Ca^2+^ into the cytoplasm along with RyRs (Figure 1) (Nixon et al., 1994). The SR is also the location that controls excitation–contraction coupling in cardiac myocytes (Li et al., 2019). When Ca^2+^ homeostasis becomes dysfunctional, toxic misfolded proteins can accumulate and cause ER stress. Ca^2+^ overload has been proposed to cause protein unfolding and activation of the ER stress response (Wiersma et al., 2017). ER stress has been directly implicated in the progression of atrial fibrillation (AF) (Wiersma et al., 2017).

The ER function comprises protein synthesis, protein folding, Ca^2+^ signaling, and proteostasis during conditions of stress. The ER is the site where proteins imported from cytosolic ribosomes are folded and modified. Proteins that are processed in the ER make up one-third of all synthesized proteins, and they are vital for cardiomyocyte function (Blackwood et al., 2019). A crucial aspect of protein folding is the formation of disulfide bonds, which is catalyzed by combining the protein disulfide isomerase and ER oxidoreductin-1 (PDI-Ero1) complex and an oxidized folding environment (Tu and Weissman, 2002; Araki et al., 2013). ROS is generated as a byproduct of disulfide bond formation during oxidative protein folding, which must be eliminated to maintain ER proteostasis. Any disturbance in redox homeostasis results in ER stress and activation of the UPR^ER^ response (Figure 2) (Zhang et al., 2019).

The UPR^ER^, like the UPR^mt^, works in an attempt to mitigate ER stress through three ER membrane-embedded sensors: protein kinase-like ER kinase (PERK), activating transcription factor 6 (ATF6), and inositol-requiring kinase 1 (IRE1) (Senft and Ronai, 2015). The activation of UPR^ER^ is regulated by the protein BiP, which inactivates the three sensors by binding to them. Binding immunoglobulin protein or heat shock protein family A member 5 (BiP/HSPA5) has a high affinity for misfolded proteins and, in response to their accumulation, will dissociate from PERK, ATF6, and IRE1 (Bertolotti et al., 2000). BiP will then act as a chaperone for the misfolded proteins, while the sensors can activate the three prongs of the ER stress response (Bettigole and Glimcher, 2015). Once ER stress is alleviated, BiP rapidly binds to the sensors again (Bertolotti et al., 2000).

ATF6 acts as a transcription factor via its N-terminus once activated through sites 1 and 2 proteolysis in the Golgi apparatus (Figure 2) (Haze et al., 1999). It works to induce genes in the protein-folding machinery that were previously not thought to play a role in protein folding (Jin et al., 2017). For instance, one study showed that ATF6 had antioxidant properties as it induced catalase activity during I/R conditions. Catalase can neutralize ROS generated as a byproduct of protein folding and protect the heart from I/R injury (Jin et al., 2017). ATF6 has been proposed as a pharmacological therapy since studies showed that selective activation of ATF6 can reduce reperfusion damage and preserve cardiac function (Blackwood et al., 2019). IRE1 activates its ER stress response by splicing X-box-binding protein 1 (Xbp1) mRNA, allowing Xbp1 to become an active transcription factor in the nucleus (Yoshida et al., 2001). Xbp1 contributes to mitigating ER stress by transcribing chaperones and proteins that assist with protein degradation (Lee et al., 2005). It also has transcriptional targets that encompass lipid metabolism (Lee et al., 2005), cellular differentiation (Acosta-Alvear et al., 2007), and elongation of the secretory protein apparatus (Shaffer et al., 2004). In a mouse model of HF with preserved ejection fraction, there was deficiency of IRE1 and Xbp1. However, restoration of Xbp1 ameliorated the phenotype in a disorder with no effective clinical therapies (Schiattarella et al., 2019). PERK acts similarly to its function in the UPR^mt^ by phosphorylating translation factor eIF2α, which causes massive downregulation of protein translation (Harding et al., 2000). This results in preferential UPR^ER^-related protein translation with a global reduction in overall protein translation. ATF4 is a protein that is preferentially translated via this pathway, where it acts as a potent activator of proapoptotic factor CHOP (Dey et al., 2010; Teske et al., 2013). CHOP may be beneficial in mild cases of cardiac ER stress, but during conditions of prolonged ER stress, it is likely harmful (Fu et al., 2010). CHOP-deficient mice had attenuated hypertrophy signs and cardiac dysfunction compared to wild-type mice, suggesting that CHOP could be implicated in the development of cardiac pathology (Fu et al., 2010). Moreover, PERK was cardioprotective in conditions of pressure overload-induced congestive HF (Liu et al., 2014). These studies indicate the complexity of the UPR^ER^ and showcases that there are instances where it is adaptive, and other cases where it is maladaptive (Arrieta et al., 2020). Hence, mild and short-lived cardiac ER stress may be beneficial to clear unfolded, misfolded, or aged proteins to maintain proper cardiac function. Prolonged ER stress that cannot be quelled, on the other hand, may be detrimental as it can result in apoptosis of cardiac cells. Since cardiomyocytes are terminally differentiated and cannot be largely replenished, excessive apoptosis will lead to cardiac dysfunction resulting in HF (Fang et al., 2019).

## Mitochondria and Sarcoplasmic Reticulum Interactions in Cardiac Physiology

The mitochondrion and SR have numerous interactions with each other, which are necessary for healthy cardiac function. Mitochondria are spatially and functionally organized in close contact with the SR. They are tightly associated in areas that support lipid and protein transfer but are more detached in areas of Ca^2+^ delivery (Csordás et al., 2010). This spatial organization contributes to the mitochondrial uptake of Ca^2+^ release from the SR via IP_3_Rs, which is essential for mitochondrial ATP production (Figure 1) (Nixon et al., 1994). Ca^2+^ transfer between these two organelles is regulated by MFN2 (De Brito and Scorrano, 2008). Dysfunction of MFN2 and other proteins found in the MAM can alter Ca^2+^ signaling, leading to aberrant signaling that promotes cardiovascular pathogenesis (Chen et al., 2012).

Ca^2+^ is also imperative to excitation–contraction coupling in cardiomyocytes, and research has attempted to elucidate whether mitochondria contribute to cytosolic Ca^2+^ regulation in contracting cardiac cells (Affolter et al., 1976). Recent evidence indicates that mitochondria participate in cardiomyocyte calcium dynamics by interacting with the SR to regulate beat-to-beat phasic calcium cycling. It is known that with every cardiac cycle, Ca^2+^ influx and efflux in the mitochondria occurs, respectively, via the MCU and the Na/Ca^2+^ exchanger (NCLX); nevertheless, the involvement of these processes in regulating excitation–contraction coupling remain largely debated. Recently, the ablation of the mitochondrial NCLX has supported the notion that indeed mitochondrial calcium regulation is vital and participates in cytosolic calcium levels and arrhythmia generation (Luongo et al., 2017). In the following sections, we will briefly describe the role of mito–SR interactions in cardiac hypertrophy, myocardial I/R injury, AF, and diabetic and inherited cardiomyopathy.

### Cardiac Hypertrophy and Heart Failure

SR–mitochondria Ca^2+^ dysregulation is thought to be a significant driver in the pathogenesis of cardiac hypertrophy and HF. This could be due to both mitochondrial Ca^2+^ overload and Ca^2+^ deficiency. Mitochondrial Ca^2+^ overload has been shown to promote mPTP opening and ROS generation. In conditions of augmented Ca^2+^ intake, increased ETC activity results in electron leakage and superoxide formation (Zoccarato et al., 2004). The increase in ROS production can lead to posttranslational modification of RyR2, causing the channel to become leaky (Santulli et al., 2015). This results in upregulation of calcium uptake by the mitochondria causing excessive mitochondrial fragmentation, reduction in size, increased permeability of the mPTP pore, and apoptosis initiation. This positive feedback loop of Ca^2+^ influx causes severe mitochondrial loss of function and HF (Santulli et al., 2015). On the other hand, mitochondrial Ca^2+^ deficiency can occur due to increased cytosolic levels of Na^+^. Increased Na^+^ levels cause increased activity of NCLX, resulting in Ca^2+^ efflux from the mitochondria. Reduced Ca^2+^ levels are thought to inhibit TCA cycle activity and increase H_2_O_2_ levels due to impaired antioxidant capabilities via a reduction in nicotinamide adenine dinucleotide phosphate (NADPH) from slower TCA cycle rates. Furthermore, H_2_O_2_ can increase Na^+^ current and influx into the mitochondria, amplifying this detrimental feedback loop (Kohlhaas et al., 2010). Inhibition of the NCLX restored Ca^2+^ levels, maintaining mitochondrial redox potential, and replenishing energy supply (Liu and Rourke, 2008). This mechanism is promising in the development of novel pharmacological strategies to treat cardiac hypertrophy and HF.

Excessive adrenergic stimulation can increase the distance between SR–mitochondrial contacts leading to mitochondrial fragmentation, impaired function, and cardiac hypertrophy (Gutiérrez et al., 2014). Norepinephrine increases the distance between the SR and mitochondria in cardiomyocytes, causing inefficient Ca^2+^ transfer (Gutiérrez et al., 2014). The mechanism by which fragmentation occurs is through an increase in Ca^2+^ and calcineurin activation. Calcineurin promotes DRP1-dependent constriction of the mitochondria, whereas the downregulation of calcineurin was shown to inhibit hypertrophy (Pennanen et al., 2014). In addition to alteration in calcineurin signaling, these hypertrophic hearts were also found to have reduced levels of MFN2, resulting in less tethering between the SR and mitochondria and mitochondrial fission (Pennanen et al., 2014). In summary, there is evidence that dysregulation of Ca^2+^ signaling and impairment of mitochondrial dynamics participate in the development of hypertrophy and HF and may be used in the future to develop novel treatment strategies.

### Myocardial Infarction and Ischemia/Reperfusion Injury

An MI is defined as the cardiomyocyte death that occurs as a result of prolonged ischemia, while I/R injury describes the tissue damage when perfusion is restored after an ischemic event. Dysfunction in Ca^2+^ transfer and contacts between mitochondria and SR can contribute to cardiac I/R injury. The upregulation of VDAC1, GRP75, and IP_3_R channel complex during I/R can lead to Ca^2+^ overload, thus triggering the opening of the mPTP. Once the mPTP pore opens, oxidative stress, mitochondrial swelling, and release of cytochrome c into the cytoplasm ensue leading to cell death (Zhu et al., 2018). Independent downregulation of VDAC1, GRP75, IP_3_R, Mfn2, and cyclophilin D (CypD) (mPTP regulator) decreased Ca^2+^ transfer and reduced cell death after I/R injury (Paillard et al., 2013). During I/R, GSK3β activates IP_3_R channels via phosphorylation. SB21 inhibition of GSK3β reduces IP_3_R channel opening and subsequent mPTP-mediated cell death (Gomez et al., 2016). Administration of sulodexide, a glycosaminoglycan that inhibits ER stress, reduces I/R-induced cellular apoptosis, leading to cardioprotection in murine models (Shen et al., 2019). Activation of the phosphoinositide 3-kinase/protein kinase B (PI_3_K/Akt) pathway causes downstream increase in Bcl-2, an antiapoptotic factor, and a decrease in Bax, a proapoptotic factor, thus reducing apoptosis (Shen et al., 2019). It is paramount to fully understand the role of mito–SR communication in ischemic cardiac injury as it may provide novel and unexplored mechanisms that can be explored for new pharmacological interventions.

### Atrial Fibrillation

ER stress-induced cardiac remodeling, Ca^2+^ overload, and excessive autophagy are the main drivers of AF. ER stress can occur through deficiency of chaperone proteins, such as HSPA5, whereas alleviation of ER stress can be achieved with the addition of 4PBA, a chemical chaperone. ER stress has also been found to activate mitochondrial apoptosis via the mitogen-activated protein kinase (MAPK) pathway (Shi et al., 2015). Ca^2+^ overload in models of AF has been found to occur through multiple mechanisms. One study using murine models found that the oxidation of RyR2 channels increased intracellular Ca^2+^ release to excessive levels (Xie et al., 2015). Another study using HL-1 atrial cardiomyocytes found that MCU-mediated Ca^2+^ influx enhanced tachypacing-induced mitochondrial dysfunction (Wiersma et al., 2019). Inhibition of this channel via treatment with Ru360 prevented devastating mitochondrial changes. The same study found aberrant ATP levels in models of AF and overexpression of the chaperone HSP60, mitochondrial fragmentation, cardiac remodeling, and contractile dysfunction (Wiersma et al., 2019). Mitochondrial dysfunction is pivotal in the development of AF as it is thought that structural mitochondrial changes exist before AF onset, but AF exacerbates mitochondrial functional impairment, thus promoting a positive feedback loop (Wiersma et al., 2019).

### Diabetic and Inherited Cardiomyopathy

ER stress and alterations in the interactions between the ER and mitochondria are crucial in the development of both diabetic and inherited cardiomyopathy. The pathogenesis of diabetic cardiomyopathy stems from impaired insulin signaling resulting in the metabolic switch from glucose metabolism to fatty acid oxidation. There is also decreased activity and presence of β-oxidation enzymes (Ljubkovic et al., 2019). This results in the development of metabolic stress and impaired functional cardiomyocyte efficiency (Ljubkovic et al., 2019). Increased mPTP Ca^2+^ sensitivity and intrinsic caspase-9 signaling are observed in human diabetic cardiomyocytes, presumably due to long-term metabolic stress, leading to an overproduction of mitochondrial ROS (Anderson et al., 2011). In human diabetic heart tissue, there were elevated levels of UPR^ER^ proteins CHOP and GRP78, linking cardiac diabetes to ER stress processes (Ljubkovic et al., 2019). Compromised cardiac contractility is another feature of diabetic cardiomyopathy, which has been linked to defective Ca^2+^ signaling linked to erratic RyR2 channel behavior (Yaras et al., 2005). ER stress-mediated apoptosis and ROS production in conditions of hyperglycemia have been reported in rats with diabetic cardiomyopathy (Yang et al., 2017). Administration of exogenous H_2_S inhibited apoptosis and ER stress via the suppression of hyperglycemia and MFN2-induced oxidative stress (Yang et al., 2017). There is limited evidence linking MERCs and pathogenesis of diabetic CM, but one study found that FUNDC1—an outer mitochondrial membrane protein—levels are elevated in diabetic patients compared to those in nondiabetic patients. It was concluded in the study that diabetes facilitates Fundc1-mediated MERC formation, and inhibition of Fundc1 could be a potential therapy for diabetic CM (Wu et al., 2019).

Inherited cardiomyopathy comprises around 50% of all cases of cardiomyopathy, with the most prevalent forms being dilated cardiomyopathy (DCM) and hypertrophic cardiomyopathy (HCM) (Towbin, 2014; Sacchetto et al., 2019). The most common disease-causing mutation in HCM comes from genes encoding sarcomeric proteins (Singh et al., 2017). The Mybpc3 gene encodes for cardiac myosin-binding protein C (cMyBP-C). A mutation of the Mybpc3 gene in human tissue, and mouse knock-in models supported the notion that autophagy is impaired, whereas activation of autophagy resulted in amelioration of cardiomyopathy (Singh et al., 2017). In DCM patients with S143P mutation in the lamin A/C gene, there was activation of the UPR^ER^ system as observed by increased ER stress markers (GRP78, IRE1, and ATF6) (Ortega et al., 2014; West et al., 2016). As intraorganellar membrane communication plays a role in many of the processes mentioned above, further understanding of these mechanisms may aid in the development of new pharmacological targets that will, at the very least, delay pathological outcomes.

## Therapeutic Targets for Mitochondrial–Endoplasmic Reticulum Interactions

### Lifestyle Interventions

Participation in secondary prevention lifestyle changes such as exercise training and caloric restriction has been shown to protect against cardiac risk factors and improve the quality of life of individuals (Clark et al., 2005). Swimming exercise in aged mice suppressed ER stress responses delaying ROS-mediated cell damage by enhancing antioxidation mechanisms via increased superoxide dismutase (Chang et al., 2020). Resistance exercise and aerobic exercise significantly decreased the expression of ER stress markers (CHOP, eIF2α, and PERK) (Kim et al., 2018). Moreover, exercise training was further potentiated when performed concurrently with a lower caloric intake (Kim et al., 2017). This agrees with results showing that caloric restriction delays proteostasis collapse that occurs with cardiac pathogenesis by maintaining robust UPR^ER^ activity (Matai et al., 2019).

### Pharmacological Interventions

Pharmacologic interventions are essential in cases where lifestyle modifications are unable to slow the progression of cardiac disease. They work to restore the balance of the ER–mitochondrial interactions and reduce ER and mitochondrial maladaptive outcomes. The use of 4BPA, a chemical chaperone, which relieves ER stress by reducing misfolded protein aggregation, has been contemplated for the use in cardiac pathologies. 4BPA is available to treat urea cycle disorders and is undergoing human clinical trials for neurological protein misfolding disorders. Thus, this strategy is also under consideration for cardiac diseases, particularly for AF, where autophagosome formation appears to be a hallmark (Wiersma et al., 2017). Another option for intervention is SIRT1, a deacetylase, which has been found to increase beneficial autophagy and decrease ER stress-induced cell death in cardiomyocytes. It promotes autophagy via indirect activation of the eukaryotic elongation factor 2 kinase (eEFK2/eEF2) pathway, possibly through regulation of the acetylation state of eIF2α (Pires Da Silva et al., 2020). The role of beta-blockers in alleviating ER stress in conditions of hypertrophy and HF are also being studied. Beta blockade inhibits beta-adrenergic hyperactivation and drastically reduces ER-mediated apoptosis in cardiomyocytes of hypertrophic and failing hearts (Ni et al., 2011). Last, the administration of taurine, a conditionally essential amino acid, has been implicated in the downregulation of mitochondrial and UPR-dependent cell apoptosis as well as ER stress markers (Yang et al., 2013).

## Conclusions

Significant scientific achievements in the twenty-first century have promoted novel pharmacological interventions to maintain cardiac function, yet cardiac disease remains a top cause of death worldwide. Interactions between mitochondria and ER are essential for a healthy myocardium as these processes participate in energy production, apoptosis, ROS management, protein folding, and Ca^2+^ signaling. Disruption in ER or mitochondrial function can play a role in development in hypertrophy, heart failure, myocardial I/R injury, AF, HCM, DCM, and diabetic cardiomyopathy. Several of these pathologies stem from common MERC alterations, but it is unknown why these changes result in different pathologies. Studies further delineating precise mechanisms that regulate intra-organellar membrane communication hold promise to unravel novel therapies that conserve cardiac function by maintaining the ER and mitochondria homeostatic balance and overall cell health.

## Author Contributions

VK, AL, and PS reviewed the literature, drafted, and edited the manuscript. VK and JS edited the manuscript and prepared illustrations. AR, SK, AD, and PS reviewed the article and edited the manuscript. All authors contributed to the article and approved the submitted version.

## Conflict of Interest

The authors declare that the research was conducted in the absence of any commercial or financial relationships that could be construed as a potential conflict of interest.

## References

[B1] Acosta-Alvear D. Zhou Y. Blais A. Tsikitis M. Lents N. H. Arias C.. (2007). XBP1 controls diverse cell type- and condition-specific transcriptional regulatory networks. Mol. Cell 27, 53–66. 10.1016/j.molcel.2007.06.011 17612490

[B2] Adachi Y. Itoh K. Yamada T. Cerveny K. L. Suzuki T. L. Macdonald P.. (2016). Coincident phosphatidic acid interaction restrains Drp1 in mitochondrial division. Mol. Cell 63, 1034–1043. 10.1016/j.molcel.2016.08.013 27635761 PMC5028122

[B3] Affolter H. Chiesi M. Dabrowska R. Carafoli E. (1976). Calcium regulation in heart cells: the interaction of mitochondrial and sarcoplasmic reticulum with troponin-bound *calcium*. Eur. J. Biochem. 67, 389–396. 10.1111/j.1432-1033.1976.tb10703.x 964249

[B4] Alavian K. N. Dworetzky S. I. Bonanni L. Zhang P. Sacchetti S. Li H.. (2015). The mitochondrial complex V - associated large-conductance inner membrane current is regulated by cyclosporine and dexpramipexole. Mol. Pharmacol. 87, 1–8. 10.1124/mol.114.095661 25332381 PMC4279080

[B5] Aldridge J. E. Horibe T. Hoogenraad N. J. (2007). Discovery of genes activated by the Mitochondrial Unfolded Protein Response (mtUPR) and cognate promoter elements. PLoS ONE 2:e874. 10.1371/journal.pone.0000874 17849004 PMC1964532

[B6] Allwood M. A. Kinobe R. T. Ballantyne L. Romanova N. Melo L. G. Ward C. A.. (2014). Heme oxygenase-1 overexpression exacerbates heart failure with aging and pressure overload but is protective against isoproterenol-induced cardiomyopathy in mice. Cardiovasc. Pathol. 23, 231–237. 10.1016/j.carpath.2014.03.007 24813593

[B7] Amchenkova A. A. Bakeeva L. E. Chentsov Y. S. Skulachev V. P. Zorov D. B. (1988). Coupling membranes as energy-transmitting cables. I. Filamentous mitochrondia in fibroblasts and mitochondrial clusters in cardiomyocytes. J. Cell Biol. 107, 481–495. 10.1083/jcb.107.2.481 3417757 PMC2115217

[B8] Anderson E. J. Rodriguez E. Anderson C. A. Thayne K. Chitwood W. R. Kypson A. P. (2011). Increased propensity for cell death in diabetic human heart is mediated by mitochondrial-dependent pathways. Am. J. Physiol. Hear Circ. Physiol. 300, H118–H124. 10.1152/ajpheart.00932.2010 21076025 PMC3023249

[B9] Andrienko T. N. Picht E. Bers D. M. (2009). Mitochondrial free calcium regulation during sarcoplasmic reticulum calcium release in rat cardiac myocytes. J. Mol. Cell. Cardiol. 46, 1027–1036. 10.1016/j.yjmcc.2009.03.015 19345225 PMC2683203

[B10] Araki K. Iemura S. Kamiya Y. Ron D. Kato K. Natsume T.. (2013). Ero1-α and pdis constitute a hierarchical electron transfer network of endoplasmic reticulum oxidoreductases. J. Cell Biol. 202, 861–874. 10.1083/jcb.201303027 24043701 PMC3776355

[B11] Arbustini E. Diegoli M. Fasani R. Grasso M. Morbini P. Banchieri N.. (1998). Mitochondrial DNA mutations and mitochondrial abnormalities in dilated cardiomyopathy. Am. J. Pathol. 153, 1501–1510. 10.1016/S0002-9440(10)65738-0 9811342 PMC1853408

[B12] Arrieta A. Blackwood E. A. Stauffer W. T. Glembotski C. C. (2020). Integrating ER and mitochondrial proteostasis in the healthy and diseased heart. Front. Cardiovasc. Med. 6:193. 10.3389/fcvm.2019.00193 32010709 PMC6974444

[B13] Baker B. M. Nargund A. M. Sun T. Haynes C. M. (2012). Protective coupling of mitochondrial function and protein synthesis via the eIF2α kinase GCN-2. PLoS Genet. 8:se1002760. 10.1371/journal.pgen.1002760 22719267 PMC3375257

[B14] Balaban R. S. Kantor H. L. Katz L. A. Briggs R. W. (1986). Relation between work and phosphate metabolite in the *in vivo* paced mammalian *heart*. Science 232, 1121–1123. 10.1126/science.3704638 3704638

[B15] Ban T. Ishihara T. Kohno H. Saita S. Ichimura A. Maenaka K.. (2017). Molecular basis of selective mitochondrial fusion by heterotypic action between OPA1 and cardiolipin. Nat. Cell Biol. 19, 856–863. 10.1038/ncb3560 28628083

[B16] Bark C. J. (1980). Mitochondrial creatine kinase: a poor prognostic sign. JAMA 243, 2058–2060. 10.1001/jama.1980.03300460040024 7373746

[B17] Bertolotti A. Zhang Y. Hendershot L. M. Harding H. P. Ron D. (2000). Dynamic interaction of BiP and ER stress transducers in the unfolded-protein *response*. Nat. Cell Biol. 2, 326–332. 10.1038/35014014 10854322

[B18] Bettigole S. E. Glimcher L. H. (2015). Endoplasmic reticulum stress in immunity. Annu. Rev. Immunol. 33, 107–138. 10.1146/annurev-immunol-032414-112116 25493331

[B19] Bittremieux M. Parys J. B. Pinton P. Bultynck G. (2016). ER functions of oncogenes and tumor suppressors: modulators of intracellular Ca2+ signaling. Biochim. Biophys. Acta Mol. Cell Res. 1863, 1364–1378. 10.1016/j.bbamcr.2016.01.002 26772784

[B20] Blackwood E. A. Azizi K. Thuerauf D. J. Paxman R. J. Plate L. Kelly J. W.. (2019). Pharmacologic ATF6 activation confers global protection in widespread disease models by reprograming cellular proteostasis. Nat. Commun. 10:187. 10.1038/s41467-018-08129-2 30643122 PMC6331617

[B21] Boveris A. Cadenas E. Stoppani A. O. (1976). Role of ubiquinone in the mitochondrial generation of hydrogen peroxide. Biochem. J. 156, 435–444. 10.1042/bj1560435 182149 PMC1163765

[B22] Braschi E. Goyon V. Zunino R. Mohanty A. Xu L. McBride H. M. (2010). Vps35 mediates vesicle transport between the mitochondria and peroxisomes. Curr. Biol. 20, 1310–1315. 10.1016/j.cub.2010.05.066 20619655

[B23] Bravo-Sagua R. Parra V. Ortiz-Sandoval C. Navarro-Marquez M. Rodríguez A. E. Diaz-Valdivia N. (2019). Caveolin-1 impairs PKA-DRP1-mediated remodelling of ER–mitochondria communication during the early phase of ER stress. Cell Death Differ. 26, 1195–1212. 10.1038/s41418-018-0197-1 30209302 PMC6748148

[B24] Bustillo-Zabalbeitia I. Montessuit S. Raemy E. Basañez G. Terrones O. Martinou J. C. (2014). Specific interaction with cardiolipin triggers functional activation of dynamin-related protein 1. PLoS ONE 9:102738. 10.1371/journal.pone.0102738 25036098 PMC4103857

[B25] Carroll J. He J. Ding S. Fearnley I. M. Walker J. E. (2019). Persistence of the permeability transition pore in human mitochondria devoid of an assembled ATP synthase. Proc. Natl. Acad. Sci. U.S.A. 116, 12816–12821. 10.1073/pnas.1904005116 31213546 PMC6601249

[B26] Chang C. R. Blackstone C. (2007). Cyclic AMP-dependent protein kinase phosphorylation of Drp1 regulates its GTPase activity and mitochondrial morphology. J. Biol. Chem. 282, 21583–21587. 10.1074/jbc.C700083200 17553808

[B27] Chang P. Zhang X. Zhang M. Li G. Hu L. Zhao H.. (2020). Swimming exercise inhibits myocardial ER stress in the hearts of aged mice by enhancing cGMP-PKG signaling. Mol. Med. Rep. 21, 549–556. 10.3892/mmr.2019.10864 31974605 PMC6947875

[B28] Chen H. Chan D. C. (2005). Emerging functions of mammalian mitochondrial fusion and fission. Hum. Mol. Genet. 14, 312–318. 10.1093/hmg/ddi270 16244327

[B29] Chen Y. Csordás G. Jowdy C. Schneider T. G. Csordás N. Wang W.. (2012). Mitofusin 2-containing mitochondrial-reticular microdomains direct rapid cardiomyocyte bioenergetic responses via interorganelle Ca2+ crosstalk. Circ. Res. 111, 863–875. 10.1161/CIRCRESAHA.112.266585 22777004 PMC3444672

[B30] Chistiakov D. A. Shkurat T. P. Melnichenko A. A. Grechko A. V. Orekhov A. N. (2018). The role of mitochondrial dysfunction in cardiovascular disease: a brief review. Ann. Med. 50, 121–127. 10.1080/07853890.2017.1417631 29237304

[B31] Choi S. Y. Huang P. Jenkins G. M. Chan D. C. Schiller J. Frohman M. A. (2006). A common lipid links Mfn-mediated mitochondrial fusion and SNARE-regulated exocytosis. Nat. Cell Biol. 8, 1255–1262. 10.1038/ncb1487 17028579

[B32] Cipolat S. De Brito O. M. Dal Zilio B. Scorrano L. (2004). OPA1 requires mitofusin 1 to promote mitochondrial fusion. Proc. Natl. Acad. Sci. U.S.A. 101, 15927–15932. 10.1073/pnas.0407043101 15509649 PMC528769

[B33] Clark A. M. Hartling L. Vandermeer B. McAlister F. A. (2005). Meta-analysis: secondary prevention programs for patients with coronary *artery*. Ann. Intern. Med. 143, 659–672. 10.7326/0003-4819-143-9-200511010-00010 16263889

[B34] Coronado M. Fajardo G. Nguyen K. Zhao M. Kooiker K. Jung G.. (2018). Physiological mitochondrial fragmentation is a normal cardiac adaptation to increased energy demand. Circ. Res. 122, 282–295. 10.1161/CIRCRESAHA.117.310725 29233845 PMC5775047

[B35] Csordás G. Várnai P. Golenár T. Roy S. Purkins G. Schneider T. G.. (2010). Imaging interorganelle contacts and local calcium dynamics at the ER-mitochondrial interface. Mol. Cell 39, 121–132. 10.1016/j.molcel.2010.06.029 20603080 PMC3178184

[B36] De Brito O. M. Scorrano L. (2008). Mitofusin 2 tethers endoplasmic reticulum to mitochondria. Nature 456, 605–610. 10.1038/nature07534 19052620

[B37] De Giorgi F. Lartigue L. Bauer M. K. A. Schubert A. Grimm S. Hanson G. T.. (2002). The permeability transition pore signals apoptosis by directing Bax translocation and multimerization. FASEB J. 16, 607–609. 10.1096/fj.01-0269fje 11919169

[B38] De la Fuente S. Sheu S. S. (2019). SR-mitochondria communication in adult cardiomyocytes: a close relationship where the Ca 2+ has a lot to say. Arch. Biochem. Biophys. 663, 259–268. 10.1016/j.abb.2019.01.026 30685253 PMC6377816

[B39] Deepa S. S. Bhaskaran S. Ranjit R. Qaisar R. Nair B. C. Liu Y.. (2016). Down-regulation of the mitochondrial matrix peptidase ClpP in muscle cells causes mitochondrial dysfunction and decreases cell proliferation. Free Radic. Biol. Med. 91, 281–292. 10.1016/j.freeradbiomed.2015.12.021 26721594 PMC5584630

[B40] Dey S. Baird T. D. Zhou D. Palam L. R. Spandau D. F. Wek R. C. (2010). Both transcriptional regulation and translational control of ATF4 are central to the integrated stress response. J. Biol. Chem. 285, 33165–33174. 10.1074/jbc.M110.167213 20732869 PMC2963398

[B41] Dorn G. W. (2010). Mitochondrial pruning by nix and BNip3: an essential function for cardiac-expressed death factors. J. Cardiovasc. Transl. Res. 3, 374–383. 10.1007/s12265-010-9174-x 20559783 PMC2900478

[B42] Drago I. De Stefani D. Rizzuto R. Pozzan T. (2012). Mitochondrial Ca2+ uptake contributes to buffering cytoplasmic Ca2+ peaks in cardiomyocytes. Proc. Natl. Acad. Sci. U.S.A. 109, 12986–12991. 10.1073/pnas.1210718109 22822213 PMC3420165

[B43] Dyck J. R. B. Cheng J. F. Stanley W. C. Barr R. Chandler M. P. Brown S. (2004). Malonyl coenzyme a decarboxylase inhibition protects the ischemic heart by inhibiting fatty acid oxidation and stimulating glucose oxidation. Circ. Res. 94, e78–e84. 10.1161/01.res.0000129255.19569.8f 15105298

[B44] Eisner V. Cupo R. R. Gao E. Csordás G. Slovinsky W. S. Paillard M.. (2017). Mitochondrial fusion dynamics is robust in the heart and depends on calcium oscillations and contractile activity. Proc. Natl. Acad. Sci. U.S.A. 114, E859–E868. 10.1073/pnas.1617288114 28096338 PMC5293028

[B45] Fang X. Wang H. Han D. Xie E. Yang X. Wei J.. (2019). Ferroptosis as a target for protection against cardiomyopathy. Proc. Natl. Acad. Sci. U.S.A. 116, 2672–2680. 10.1073/pnas.1821022116 30692261 PMC6377499

[B46] Fiorese C. J. Schulz A. M. Lin Y. F. Rosin N. Pellegrino M. W. Haynes C. M. (2016). The transcription factor ATF5 mediates a mammalian mitochondrial UPR. Curr. Biol. 26, 2037–2043. 10.1016/j.cub.2016.06.002 27426517 PMC4980197

[B47] Förstermann U. Xia N. Li H. (2017). Roles of vascular oxidative stress and nitric oxide in the pathogenesis of atherosclerosis. Circ. Res. 120, 713–735. 10.1161/CIRCRESAHA.116.309326 28209797

[B48] Frank S. Gaume B. Bergmann-Leitner E. S. Leitner W. W. Robert E. G. Catez F.. (2001). The role of dynamin-related protein 1, a mediator of mitochondrial fission, in apoptosis. Dev. Cell 1, 515–525. 10.1016/S1534-5807(01)00055-7 11703942

[B49] Franzini-Armstrong C. (2007). ER-mitochondria communication. How privileged? Physiology 22, 261–268. 10.1152/physiol.00017.2007 17699879

[B50] Friedman J. R. DiBenedetto J. R. West M. Rowland A. A. Voeltz G. K. (2013). Endoplasmic reticulum-endosome contact increases as endosomes traffic and mature. Mol. Biol. Cell 24, 1030–1040. 10.1091/mbc.E12-10-0733 23389631 PMC3608491

[B51] Friedman J. R. Lackner L. L. West M. DiBenedetto J. R. Nunnari J. Voeltz G. K. (2011). ER tubules mark sites of mitochondrial division. Science 334, 358–362. 10.1126/science.1207385 21885730 PMC3366560

[B52] Fu H. Y. Okada K. I. Liao Y. Tsukamoto O. Isomura T. Asai M.. (2010). Ablation of C/EBP homologous protein attenuates endoplasmic reticulum-mediated apoptosis and cardiac dysfunction induced by pressure overload. Circulation 122, 361–369. 10.1161/CIRCULATIONAHA.109.917914 20625112

[B53] Gadicherla A. K. Wang N. Bulic M. Agullo-Pascual E. Lissoni A. De Smet M.. (2017). Mitochondrial Cx43 hemichannels contribute to mitochondrial calcium entry and cell death in the heart. Basic Res. Cardiol. 112:27. 10.1007/s00395-017-0618-1 28364353

[B54] Galluzzi L. Baehrecke E. H. Ballabio A. Boya P. Bravo-San Pedro J. M. Cecconi F.. (2017). Molecular definitions of autophagy and related processes. EMBO J. 36, 1811–1836. 10.15252/embj.201796697 28596378 PMC5494474

[B55] Gálvez A. S. Brunskill E. W. Marreez Y. Benner B. J. Regula K. M. Kirschenbaum L. A.. (2006). Distinct pathways regulate proapoptotic Nix and BNip3 in cardiac stress. J. Biol. Chem. 281, 1442–1448. 10.1074/jbc.M509056200 16291751

[B56] Gandre-Babbe S. Van Der Bliek A. M. (2008). The novel tail-anchored membrane protein Mff controls mitochondrial and peroxisomal fission in mammalian cells. Mol. Biol. Cell 19, 2402–2412. 10.1091/mbc.E07-12-1287 18353969 PMC2397315

[B57] Gao M. Yi J. Zhu J. Minikes A. M. Monian P. Thompson C. B.. (2019). Role of mitochondria in ferroptosis. Mol. Cell 73, 354–363.e3. 10.1016/j.molcel.2018.10.042 30581146 PMC6338496

[B58] Garbincius J. F. Luongo T. S. Elrod J. W. (2020). The debate continues – what is the role of MCU and mitochondrial calcium uptake in the heart? J. Mol. Cell. Cardiol. 143, 163–174. 10.1016/j.yjmcc.2020.04.029 32353353 PMC7938348

[B59] Gawlowski T. Suarez J. Scott B. Torres-Gonzalez M. Wang H. Schwappacher R.. (2012). Modulation of dynamin-related protein 1 (DRP1) function by increased O-linked-β-N-acetylglucosamine modification (O-GlcNAc) in cardiac myocytes. J. Biol. Chem. 287, 30024–30034. 10.1074/jbc.M112.390682 22745122 PMC3436129

[B60] Gegg M. E. Cooper J. M. Chau K. Y. Rojo M. Schapira A. H. V. Taanman J. W. (2010). Mitofusin 1 and mitofusin 2 are ubiquitinated in a PINK1/parkin-dependent manner upon induction of mitophagy. Hum. Mol. Genet. 19, 4861–4870. 10.1093/hmg/ddq419 20871098 PMC3583518

[B61] Giacomello M. Pellegrini L. (2016). The coming of age of the mitochondria-ER contact: a matter of thickness. Cell Death Differ. 23, 1417–1427. 10.1038/cdd.2016.52 27341186 PMC5072433

[B62] Gomez L. Thiebaut P. A. Paillard M. Ducreux S. Abrial M. Crola Da Silva C.. (2016). The SR/ER-mitochondria calcium crosstalk is regulated by GSK3β during reperfusion injury. Cell Death Differ. 23, 313–322. 10.1038/cdd.2015.101 26206086 PMC4716295

[B63] Gomez-Suaga P. Paillusson S. Stoica R. Noble W. Hanger D. P. Miller C. C. J. (2017). The ER-mitochondria tethering complex VAPB-PTPIP51 regulates autophagy. Curr. Biol. 27, 371–385. 10.1016/j.cub.2016.12.038 28132811 PMC5300905

[B64] Griffiths E. J. (1999). Species dependence of mitochondrial calcium transients during excitation-contraction coupling in isolated cardiomyocytes. Biochem. Biophys. Res. Commun. 263, 554–559. 10.1006/bbrc.1999.1311 10491330

[B65] Gutiérrez T. Parra V. Troncoso R. Pennanen C. Contreras-Ferrat A. Vasquez-Trincado C.. (2014). Alteration in mitochondrial Ca2+ uptake disrupts insulin signaling in hypertrophic cardiomyocytes. Cell Commun. Signal. 12:68. 10.1186/s12964-014-0068-4 25376904 PMC4234850

[B66] Harding H. P. Zhang Y. Bertolotti A. Zeng H. Ron D. (2000). Perk is essential for translational regulation and cell survival during the unfolded protein response. Mol. Cell 5, 897–904. 10.1016/S1097-2765(00)80330-5 10882126

[B67] Haynes C. M. Petrova K. Benedetti C. Yang Y. Ron D. (2007). ClpP mediates activation of a mitochondrial unfolded protein response in C. elegans. Dev. Cell 13, 467–480. 10.1016/j.devcel.2007.07.016 17925224

[B68] Haze K. Yoshida H. Yanagi H. Yura T. Mori K. (1999). Mammalian transcription factor ATF6 is synthesized as a transmembrane protein and activated by proteolysis in response to endoplasmic reticulum stress. Mol. Biol. Cell 10, 3787–3799. 10.1091/mbc.10.11.3787 10564271 PMC25679

[B69] Hernández-Alvarez M. I. Sebastián D. Vives S. Ivanova S. Bartoccioni P. Kakimoto P.. (2019). Deficient endoplasmic reticulum-mitochondrial phosphatidylserine transfer causes liver disease. Cell 177, 881–895.e17. 10.1016/j.cell.2019.04.010 31051106

[B70] Hirabayashi Y. Kwon S. K. Paek H. Pernice W. M. Paul M. A. Lee J.. (2017). ER-mitochondria tethering by PDZD8 regulates Ca2+ dynamics in mammalian neurons. Science 358, 623–630. 10.1126/science.aan6009 29097544 PMC5818999

[B71] Honrath B. Metz I. Bendridi N. Rieusset J. Culmsee C. Dolga A. M. (2017). Glucose-regulated protein 75 determines ER–mitochondrial coupling and sensitivity to oxidative stress in neuronal cells. Cell Death Discov. 3:17076. 10.1038/cddiscovery.2017.76 29367884 PMC5672593

[B72] Hoppel C. L. Tandler B. Fujioka H. Riva A. (2009). Dynamic organization of mitochondria in human heart and in myocardial disease. Int. J. Biochem. Cell Biol. 41, 1949–1956. 10.1016/j.biocel.2009.05.004 19446651 PMC3221317

[B73] Hoshino A. Mita Y. Okawa Y. Ariyoshi M. Iwai-Kanai E. Ueyama T.. (2013). Cytosolic p53 inhibits Parkin-mediated mitophagy and promotes mitochondrial dysfunction in the mouse heart. Nat. Commun. 4:2308. 10.1038/ncomms3308 23917356

[B74] Huang X. Sun L. Ji S. Zhao T. Zhang W. Xu J.. (2013). Kissing and nanotunneling mediate intermitochondrial communication in the heart. Proc. Natl. Acad. Sci. U.S.A. 110, 2846–2851. 10.1073/pnas.1300741110 23386722 PMC3581932

[B75] Ikeda Y. Shirakabe A. Maejima Y. Zhai P. Sciarretta S. Toli J.. (2015). Endogenous Drp1 mediates mitochondrial autophagy and protects the heart against energy stress. Circ. Res. 116, 264–278. 10.1161/CIRCRESAHA.116.303356 25332205

[B76] Iwasawa R. Mahul-Mellier A. L. Datler C. Pazarentzos E. Grimm S. (2011). Fis1 and Bap31 bridge the mitochondria-ER interface to establish a platform for apoptosis induction. EMBO J. 30, 556–568. 10.1038/emboj.2010.346 21183955 PMC3034017

[B77] Jackson S. L. Tong X. King R. J. Loustalot F. Hong Y. Ritchey M. D. (2018). National burden of heart failure events in the United States, 2006 to 2014. Circ. Heart Fail 11:e004873. 10.1161/CIRCHEARTFAILURE.117.004873 30562099 PMC6424109

[B78] Jin J. K. Blackwood E. A. Azizi K. Thuerauf D. J. Fahem A. G. Hofmann C.. (2017). ATF6 decreases myocardial ischemia/reperfusion damage and links ER stress and oxidative stress signaling pathways in the heart. Circ. Res. 120, 862–875. 10.1161/CIRCRESAHA.116.310266 27932512 PMC5336510

[B79] Kane L. A. Lazarou M. Fogel A. I. Li Y. Yamano K. Sarraf S. A.. (2014). PINK1 phosphorylates ubiquitin to activate parkin E3 ubiquitin ligase activity. J. Cell Biol. 205, 143–153. 10.1083/jcb.201402104 24751536 PMC4003245

[B80] Kim K. Ahn N. Jung S. (2018). Comparison of endoplasmic reticulum stress and mitochondrial biogenesis responses after 12 weeks of treadmill running and ladder climbing exercises in the cardiac muscle of middle-aged obese rats. Brazilian J. Med. Biol. Res. 51:e7508. 10.1590/1414-431X20187508 30066723 PMC6075797

[B81] Kim K. Ahn N. Jung S. Park S. (2017). Effects of intermittent ladder-climbing exercise training on itochondrial biogenesis and endoplasmic reticulum stress of the cardiac muscle in obese middle-aged rats. Korean J. Physiol. Pharmacol. 21, 633–641. 10.4196/kjpp.2017.21.6.633 29200906 PMC5709480

[B82] Knoblach B. Sun X. Coquelle N. Fagarasanu A. Poirier R. L. Rachubinski R. A. (2013). An ER-peroxisome tether exerts peroxisome population control in yeast. EMBO J. 32, 2439–2453. 10.1038/emboj.2013.170 23900285 PMC3770948

[B83] Kohlhaas M. Liu T. Knopp A. Zeller T. Ong M. F. Böhm M.. (2010). Elevated cytosolic Na+ increases mitochondrial formation of reactive oxygen species in failing cardiac myocytes. Circulation 121, 1606–1613. 10.1161/CIRCULATIONAHA.109.914911 20351235 PMC2946079

[B84] Kornmann B. Currie E. Collins S. R. Schuldiner M. Nunnari J. Weissman J. S.. (2009). An ER-mitochondria tethering complex revealed by a synthetic biology screen. Science 325, 477–481. 10.1126/science.1175088 19556461 PMC2933203

[B85] Kraus B. Cain H. (1980). Giant mitochondria in the human myocardium - morphogenesis and fate. Virchows Arch. B Cell Pathol. Incl. Mol. Pathol. 33, 77–89. 10.1007/BF02899172 6110267

[B86] Lavorato M. Iyer V. R. Dewight W. Cupo R. R. Debattisti V. Gomez L.. (2017). Increased mitochondrial nanotunneling activity, induced by calcium imbalance, affects intermitochondrial matrix exchanges. Proc. Natl. Acad. Sci. U.S.A. 114, E849–E858. 10.1073/pnas.1617788113 28096415 PMC5293110

[B87] Lazarou M. Sliter D. A. Kane L. A. Sarraf S. A. Wang C. Burman J. L.. (2015). The ubiquitin kinase PINK1 recruits autophagy receptors to induce mitophagy. Nature 524, 309–314. 10.1038/nature14893 26266977 PMC5018156

[B88] Lee A. H. Chu G. C. Iwakoshi N. N. Glimcher L. H. (2005). XBP-1 is required for biogenesis of cellular secretory machinery of exocrine glands. EMBO J. 24, 4368–4380. 10.1038/sj.emboj.7600903 16362047 PMC1356340

[B89] Lee S. Min K. T. (2018). The interface between ER and mitochondria: molecular compositions and functions. Mol. Cells 41, 1000–1007. 10.14348/molcells.2018.0438 30590907 PMC6315321

[B90] Lee Y. J. Jeong S. Y. Karbowski M. Smith C. L. Youle R. J. (2004). Roles of the mammalian mitochondrial fission and fusion mediators Fis1, Drp1, Opa1 in apoptosis. Mol. Biol. Cell 15, 5001–5011. 10.1091/mbc.E04-04-0294 15356267 PMC524759

[B91] Li J. Zhang D. Brundel B. J. J. M. Wiersma M. (2019). Imbalance of ER and mitochondria interactions: prelude to cardiac ageing and disease? Cells 8:1617. 10.3390/cells8121617 31842269 PMC6952992

[B92] Liu T. Rourke B. O. (2008). Enhancing mitochondrial Ca2+ uptake in myocytes from failing hearts restores energy supply and demand matching. Circ. Res. 103, 279–288. 10.1161/CIRCRESAHA.108.175919 18599868 PMC2711021

[B93] Liu X. Kwak D. Lu Z. Xu X. Fassett J. Wang H.. (2014). Endoplasmic reticulum stress sensor protein kinase R-like endoplasmic reticulum kinase (PERK) protects against pressure overload-induced heart failure and lung remodeling. Hypertension 64, 738–744. 10.1161/HYPERTENSIONAHA.114.03811 24958502 PMC4162806

[B94] Ljubkovic M. Gressette M. Bulat C. Cavar M. Bakovic D. Fabijanic D.. (2019). Disturbed fatty acid oxidation, endoplasmic reticulum stress, and apoptosis in left ventricle of patients with type 2 diabetes. Diabetes 68, 1924–1933. 10.2337/db19-0423 31391173

[B95] Lombardi A. A. Gibb A. A. Arif E. Kolmetzky D. W. Tomar D. Luongo T. S.. (2019). Mitochondrial calcium exchange links metabolism with the epigenome to control cellular differentiation. Nat. Commun. 10:4509. 10.1038/s41467-019-12103-x 31586055 PMC6778142

[B96] Losón O. C. Song Z. Chen H. Chan D. C. (2013). Fis1, Mff, MiD49, and MiD51 mediate Drp1 recruitment in mitochondrial fission. Mol. Biol. Cell 24, 659–667. 10.1091/mbc.E12-10-0721 23283981 PMC3583668

[B97] Luongo T. S. Lambert J. P. Gross P. Nwokedi M. Lombardi A. A. Shanmughapriya S. (2017). The mitochondrial Na+/Ca2+ exchanger is essential for Ca2+ homeostasis and viability. Nature 545, 93–97. 10.1038/nature22082 28445457 PMC5731245

[B98] Luongo T. S. Lambert J. P. Yuan A. Zhang X. Gross P. Song J.. (2015). The Mitochondrial calcium uniporter matches energetic supply with cardiac workload during stress and modulates permeability transition. Cell Rep. 12, 23–34. 10.1016/j.celrep.2015.06.017 26119731 PMC4517182

[B99] Mallilankaraman K. Cárdenas C. Doonan P. J. Chandramoorthy H. C. Irrinki K. M. Golenár T.. (2012a). MCUR1 is an essential component of mitochondrial Ca2+ uptake that regulates cellular metabolism. Nat. Cell Biol. 14, 1336–1343. 10.1038/ncb2622 23178883 PMC3511605

[B100] Mallilankaraman K. Doonan P. Cárdenas C. Chandramoorthy H. C. Müller M. Miller R.. (2012b). MICU1 is an essential gatekeeper for mcu-mediated mitochondrial Ca 2+ uptake that regulates cell survival. Cell 151, 630–644. 10.1016/j.cell.2012.10.011 23101630 PMC3486697

[B101] Marsboom G. Toth P. T. Ryan J. J. Hong Z. Wu X. Fang Y. H.. (2012). Dynamin-related protein 1-mediated mitochondrial mitotic fission permits hyperproliferation of vascular smooth muscle cells and offers a novel therapeutic target in pulmonary hypertension. Circ. Res. 110, 1484–1497. 10.1161/CIRCRESAHA.111.263848 22511751 PMC3539779

[B102] Martinus R. D. Garth G. P. Webster T. L. Cartwright P. Naylor D. J. Høj P. B.. (1996). Selective induction of mitochondrial chaperones in response to loss of the mitochondrial genome. Eur. J. Biochem. 240, 98–103. 10.1111/j.1432-1033.1996.0098h.x 8797841

[B103] Matai L. Sarkar G. C. Chamoli M. Malik Y. Kumar S. S. Rautela U.. (2019). Dietary restriction improves proteostasis and increases life span through endoplasmic reticulum hormesis. Proc. Natl. Acad. Sci. U.S.A. 116, 17383–17392. 10.1073/pnas.1900055116 31413197 PMC6717303

[B104] Moyzis A. G. Lally N. S. Liang W. Leon L. J. Najor R. H. Orogo A. M.. (2020). Mcl-1-mediated mitochondrial fission protects against stress but impairs cardiac adaptation to exercise. J. Mol. Cell. Cardiol. 146, 109–120. 10.1016/j.yjmcc.2020.07.009 32717194 PMC7494655

[B105] Münch C. Harper J. W. (2016). Mitochondrial unfolded protein response controls matrix pre-RNA processing and translation. Nature 534, 710–713. 10.1038/nature18302 27350246 PMC4939261

[B106] Murphy E. Pan X. Nguyen T. Liu J. Holmström K. M. Finkel T. (2014). Unresolved questions from the analysis of mice lacking MCU expression. Biochem. Biophys. Res. Commun. 449, 384–385. 10.1016/j.bbrc.2014.04.144 24792186 PMC4214067

[B107] Murry C. E. Jennings R. B. Reimer K. A. (1986). Preconditioning with ischemia: a delay of lethal cell injury in ischemic myocardium. Circulation 74, 1124–1136. 10.1161/01.CIR.74.5.1124 3769170

[B108] Nargund A. M. Pellegrino M. W. Fiorese C. J. Baker B. M. Haynes C. M. (2012). Mitochondrial import efficiency of ATFS-1 regulates mitochondrial UPR activation. Science 337, 587–590. 10.1126/science.1223560 22700657 PMC3518298

[B109] National Center for Health Statistics (2018). Selected Circulatory Diseases Among Adults Aged 18 and Over, by Selected Characteristics: United States, 2018. Available online at: http://www.cdc.gov/nchs/nhis/SHS/tables.htm (accessed August 1, 2020).

[B110] Neglia D. De Caterina A. Marraccini P. Natali A. Ciardetti M. Vecoli C.. (2007). Impaired myocardial metabolic reserve and substrate selection flexibility during stress in patients with idiopathic dilated cardiomyopathy. Am. J. Physiol. Hear Circ. Physiol. 293, 3270–3278. 10.1152/ajpheart.00887.2007 17921325

[B111] Neuspiel M. Schauss A. C. Braschi E. Zunino R. Rippstein P. Rachubinski R. A.. (2008). Cargo-selected transport from the mitochondria to peroxisomes is mediated by vesicular carriers. Curr. Biol. 18, 102–108. 10.1016/j.cub.2007.12.038 18207745

[B112] Ni L. Zhou C. Duan Q. Lv J. Fu X. Xia Y.. (2011). β-AR blockers suppresses ER stress in cardiac hypertrophy and heart failure. PLoS ONE 6:e27294. 10.1371/journal.pone.0027294 22073308 PMC3206949

[B113] Nixon G. F. Mignery G. A. Somlyo A. V. (1994). Immunogold localization of inositol 1,4,5-trisphosphate receptors and characterization of ultrastructural features of the sarcoplasmic reticulum in phasic and tonic smooth *muscle*. J. Muscle Res. Cell Motil. 15, 682–700. 10.1007/BF00121075 7706424

[B114] Oettinghaus B. D'Alonzo D. Barbieri E. Restelli L. M. Savoia C. Licci M.. (2016). DRP1-dependent apoptotic mitochondrial fission occurs independently of BAX, BAK and APAF1 to amplify cell death by BID and oxidative stress. Biochim. Biophys. Acta Bioenerg. 1857, 1267–1276. 10.1016/j.bbabio.2016.03.016 26997499

[B115] Ong S. B. Kalkhoran S. B. Hernández-Reséndiz S. Samangouei P. Ong S. G. Hausenloy D. J. (2017). Mitochondrial-shaping proteins in cardiac health and disease – the long and the short of it! Cardiovasc. Drugs Ther. 31, 87–107. 10.1007/s10557-016-6710-1 28190190 PMC5346600

[B116] Ortega A. Roselló-Llet,í E. Tarazón E. Molina-Navarro M. M. Martínez-Dolz L. González-Juanatey J. R.. (2014). Endoplasmic reticulum stress induces different molecular structural alterations in human dilated and ischemic cardiomyopathy. PLoS ONE 9:e107635. 10.1371/journal.pone.0107635 25226522 PMC4166610

[B117] Osman C. Voelker D. R. Langer T. (2011). Making heads or tails of phospholipids in mitochondria. J. Cell Biol. 192, 7–16. 10.1083/jcb.201006159 21220505 PMC3019561

[B118] Otera H. Wang C. Cleland M. M. Setoguchi K. Yokota S. Youle R. J.. (2010). Mff is an essential factor for mitochondrial recruitment of Drp1 during mitochondrial fission in mammalian cells. J. Cell Biol. 191, 1141–1158. 10.1083/jcb.201007152 21149567 PMC3002033

[B119] Otsu K. Willard H. F. Khanna V. K. Zorzato F. Green N. M. MacLennan D. H. (1990). Molecular cloning of cDNA encoding the Ca2+ release channel (ryanodine receptor) of rabbit cardiac muscle sarcoplasmic reticulum. J. Biol. Chem. 265, 13472–13483. 2380170

[B120] Paillard M. Tubbs E. Thiebaut P. A. Gomez L. Fauconnier J. Da Silva C. C.. (2013). Depressing mitochondria-reticulum interactions protects cardiomyocytes from lethal hypoxia-reoxygenation injury. Circulation 128, 1555–1565. 10.1161/CIRCULATIONAHA.113.001225 23983249

[B121] Palmer J. W. Tandler B. Hoppel C. L. (1977). Biochemical Properties of Subsarcolemmal and Interfibrillar Mitochondria Isolated From Rat Cardiac Muscle. Available online at: http://www.jbc.org/ (accessed August 2, 2020). 925018

[B122] Pan X. Liu J. Nguyen T. Liu C. Sun J. Teng Y.. (2013). The physiological role of mitochondrial calcium revealed by mice lacking the mitochondrial calcium uniporter. Nat. Cell Biol. 15, 1464–1472. 10.1038/ncb2868 24212091 PMC3852190

[B123] Papanicolaou K. N. Khairallah R. J. Ngoh G. A. Chikando A. Luptak I. O'Shea K. M.. (2011). Mitofusin-2 maintains mitochondrial structure and contributes to stress-induced permeability transition in cardiac myocytes. Mol. Cell. Biol. 31, 1309–1328. 10.1128/mcb.00911-10 21245373 PMC3067905

[B124] Papanicolaou K. N. Kikuchi R. Ngoh G. A. Coughlan K. A. Dominguez I. Stanley W. C.. (2012). Mitofusins 1 and 2 are essential for postnatal metabolic remodeling in heart. Circ. Res. 111, 1012–1026. 10.1161/CIRCRESAHA.112.274142 22904094 PMC3518037

[B125] Pennanen C. Parra V. López-Crisosto C. Morales P. E. del Campo A. Gutierrez T.. (2014). Mitochondrial fission is required for cardiomyocyte hypertrophy mediated by a Ca2+-calcineurin signaling pathway. J. Cell Sci. 127, 2659–2671. 10.1242/jcs.139394 24777478 PMC4058110

[B126] Peretti D. Dahan N. Shimoni E. Hirschberg K. Lev S. (2008). Coordinated lipid transfer between the endoplasmic reticulum and the golgi complex requires the VAP proteins and is essential for Golgi-mediated transport. Mol. Biol. Cell 19, 3871–3884. 10.1091/mbc.E08-05-0498 18614794 PMC2526681

[B127] Picard M. McManus M. J. Csordás G. Várnai P. Dorn G. W. Williams D.. (2015). Trans-mitochondrial coordination of cristae at regulated membrane junctions. Nat. Commun. 6:7259. 10.1038/ncomms7259 25687472 PMC4332397

[B128] Pickles S. Vigié P. Youle R. J. (2018). Mitophagy and quality control mechanisms in mitochondrial maintenance. Curr. Biol. 28, R170–R185. 10.1016/j.cub.2018.01.004 29462587 PMC7255410

[B129] Piquereau J. Caffin F. Novotova M. Prola A. Garnier A. Mateo P.. (2012). Down-regulation of OPA1 alters mouse mitochondrial morphology, PTP function, and cardiac adaptation to pressure overload. Cardiovasc. Res. 94, 408–417. 10.1093/cvr/cvs117 22406748 PMC3863708

[B130] Pires Da Silva J. Monceaux K. Guilbert A. Gressette M. Piquereau J. Novotova M.. (2020). SIRT1 protects the heart from ER stress-induced injury by promoting eEF2K/eEF2-dependent autophagy. Cells 9:426. 10.3390/cells9020426 32059483 PMC7072417

[B131] Priesnitz C. Becker T. (2018). Pathways to balance mitochondrial translation and protein import. Genes Dev. 32, 1285–1296. 10.1101/gad.316547.118 30275044 PMC6169841

[B132] Puglielli L. Konopka G. Pack-Chung E. Ingano L. A. M. K. Berezovska O. Hyman B. T.. (2001). Acyl-coenzyme a: cholesterol acyltransferase modulates the generation of the amyloid β-peptide. Nat. Cell Biol. 3, 905–912. 10.1038/ncb1001-905 11584272

[B133] Quirós P. M. Langer T. López-Otín C. (2015). New roles for mitochondrial proteases in health, ageing and disease. Nat. Rev. Mol. Cell Biol. 16, 345–359. 10.1038/nrm3984 25970558

[B134] Rambold A. S. Kostelecky B. Elia N. Lippincott-Schwartz J. (2011). Tubular network formation protects mitochondria from autophagosomal degradation during nutrient starvation. Proc. Natl. Acad. Sci. U.S.A. 108, 10190–10195. 10.1073/pnas.1107402108 21646527 PMC3121813

[B135] Ridgway N. D. Vance D. E. (1987). Purification of phosphatidylethanolamine N-methyltransferase from rat liver. J. Biochem. 262, 17231–17239. 3680298

[B136] Riva A. Tandler B. Loffredo F. Vazquez E. Hoppel C. (2005). Structural differences in two biochemically defined populations of cardiac mitochondria. Am. J. Physiol. Hear Circ. Physiol. 289, 868–872. 10.1152/ajpheart.00866.2004 15821034

[B137] Rizzuto R. Pinton P. Carrington W. Fay F. S. Fogarty K. E. Lifshitz L. M.. (1998). Close contacts with the endoplasmic reticulum as determinants of mitochondrial Ca2+ responses. Science 280, 1763–1766. 10.1126/science.280.5370.1763 9624056

[B138] Rolland S. G. Schneid S. Schwarz M. Rackles E. Fischer C. Haeussler S.. (2019). Compromised mitochondrial protein import acts as a signal for UPRmt. Cell Rep. 28, 1659–1669.e5. 10.1016/j.celrep.2019.07.049 31412237

[B139] Roth G. A. Abate D. Abate K. H. Abay S. M. Abbafati C. Abbasi N.. (2018). Global, regional, and national age-sex-specific mortality for 282 causes of death in 195 countries and territories, 1980–2017: a systematic analysis for the global burden of disease study 2017. Lancet 392, 1736–1788. 10.1016/S0140-6736(18)32203-7 30496103 PMC6227606

[B140] Rusiñol A. E. Cui Z. Chen M. H. Vance J. E. (1994). A unique mitochondria-associated membrane fraction from rat liver has a high capacity for lipid synthesis and contains pre-Golgi secretory proteins including nascent lipoproteins. J. Biol. Chem. 269, 27494–27502. 7961664

[B141] Sacchetto C. Sequeira V. Bertero E. Dudek J. Maack C. Calore M. (2019). Metabolic Alterations in Inherited Cardiomyopathies. J. Clin. Med. 8:2195. 10.3390/jcm8122195 31842377 PMC6947282

[B142] Sala-Vila A. Navarro-Lérida I. Sánchez-Alvarez M. Bosch M. Calvo C. López J. A.. (2016). Interplay between hepatic mitochondria-associated membranes, lipid metabolism and caveolin-1 in mice. Sci. Rep. 6:27351. 10.1038/srep27351 27272971 PMC4894368

[B143] Santel A. Fuller M. T. (2001). Control of mitochondrial morphology by a human mitofusin. J. Cell Sci. 114(Pt. 5), 867–874. 11181170 10.1242/jcs.114.5.867

[B144] Santulli G. Xie W. Reiken S. R. Marks A. R. (2015). Mitochondrial calcium overload is a key determinant in heart failure. Proc. Natl. Acad. Sci. U.S.A. 112, 11389–11394. 10.1073/pnas.1513047112 26217001 PMC4568687

[B145] Schiattarella G. G. Altamirano F. Tong D. French K. M. Villalobos E. Kim S. Y.. (2019). Nitrosative stress drives heart failure with preserved ejection fraction. Nature 568, 351–356. 10.1038/s41586-019-1100-z 30971818 PMC6635957

[B146] Schlame M. Haldar D. (1993). Cardiolipin is synthesized on the matrix side of the inner membrane in rat liver mitochondria. J. Biol. Chem. 268, 74–79. 8380172

[B147] Sedova M. Dedkova E. N. Blatter L. A. (2006). Integration of rapid cytosolic Ca2+ signals by mitochondria in cat ventricular myocytes. Am. J. Physiol. Cell Physiol. 291, C840–C850. 10.1152/ajpcell.00619.2005 16723510

[B148] Senft D. Ronai Z. A. (2015). UPR, autophagy, and mitochondria crosstalk underlies the ER stress response. Trends Biochem. Sci. 40, 141–148. 10.1016/j.tibs.2015.01.002 25656104 PMC4340752

[B149] Seo B. J. Choi J. La H. Habib O. Choi Y. Hong K.. (2020). Role of mitochondrial fission-related genes in mitochondrial morphology and energy metabolism in mouse embryonic stem cells. Redox Biol. 36:101599. 10.1016/j.redox.2020.101599 32521505 PMC7286981

[B150] Shaffer A. L. Shapiro-Shelef M. Iwakoshi N. N. Lee A. H. Qian S. B. Zhao H.. (2004). XBP1, downstream of Blimp-1, expands the secretory apparatus and other organelles, and increases protein synthesis in plasma cell differentiation. Immunity 21, 81–93. 10.1016/j.immuni.2004.06.010 15345222

[B151] Shanmughapriya S. Rajan S. Hoffman N. E. Higgins A. M. Tomar D. Nemani N.. (2015). SPG7 is an essential and conserved component of the mitochondrial permeability transition pore. Mol. Cell 60, 47–62. 10.1016/j.molcel.2015.08.009 26387735 PMC4592475

[B152] Shen D. Chen R. Zhang L. Rao Z. Ruan Y. Li L.. (2019). Sulodexide attenuates endoplasmic reticulum stress induced by myocardial ischaemia/reperfusion by activating the PI3K/Akt pathway. J. Cell. Mol. Med. 23, 5063–5075. 10.1111/jcmm.14367 31120192 PMC6653332

[B153] Shi J. Jiang Q. Ding X. Xu W. Wang D. W. Chen M. (2015). The ER stress-mediated mitochondrial apoptotic pathway and MAPKs modulate tachypacing-induced apoptosis in HL-1 atrial myocytes. PLoS ONE 10:e0117567. 10.1371/journal.pone.0117567 25689866 PMC4331367

[B154] Shim E. H. Livi C. B. Rakheja D. Tan J. Benson D. Parekh V.. (2014). L-2-hydroxyglutarate: an epigenetic modifier and putative oncometabolite in renal cancer. Cancer Discov. 4, 1290–1298. 10.1158/2159-8290.CD-13-0696 25182153 PMC4286872

[B155] Shore G. C. Tata J. R. (1977). Two fractions of rough endoplasmic reticulum from rat liver. I. recovery of rapidly sedimenting endoplasmic reticulum in association with mitochondria. J. Cell Biol. 72, 714–725. 10.1083/jcb.72.3.714 838772 PMC2111030

[B156] Singh S. R. Zech A. T. L. Geertz B. Reischmann-Düsener S. Osinska H. Prondzynski M.. (2017). Activation of autophagy ameliorates cardiomyopathy in Mybpc3-targeted knockin Mice. Circ. Hear. Fail. 10:e004140. 10.1161/CIRCHEARTFAILURE.117.004140 29021349 PMC5679453

[B157] Soboll S. Brdiczka D. Jahnke D. Schmidt A. Schlattner U. Wendt S.. (1999). Octamer dimer transitions of mitochondrial creatine kinase in heart disease. J. Mol. Cell Cardiol. 31, 857–866. 10.1006/jmcc.1998.0925 10329213

[B158] Soubannier V. Rippstein P. Kaufman B. A. Shoubridge E. A. McBride H. M. (2012). Reconstitution of mitochondria derived vesicle formation demonstrates selective enrichment of oxidized cargo. PLoS ONE 7:e0052830. 10.1371/journal.pone.0052830 23300790 PMC3530470

[B159] Stone S. J. Levin M. C. Zhou P. Han J. Walther T. C. Farese R. V. (2009). The endoplasmic reticulum enzyme DGAT2 is found in mitochondria-associated membranes and has a mitochondrial targeting signal that promotes its association with mitochondria. J. Biol. Chem. 284, 5352–5361. 10.1074/jbc.M805768200 19049983 PMC2643492

[B160] Szabadkai G. Bianchi K. Várnai P. De Stefani D. Wieckowski M. R. Cavagna D.. (2006). Chaperone-mediated coupling of endoplasmic reticulum and mitochondrial Ca2+ channels. J. Cell Biol. 175, 901–911. 10.1083/jcb.200608073 17178908 PMC2064700

[B161] Szymański J. Janikiewicz J. Michalska B. Patalas-Krawczyk P. Perrone M. Ziółkowski W.. (2017). Interaction of mitochondria with the endoplasmic reticulum and plasma membrane in calcium homeostasis, lipid trafficking and mitochondrial structure. Int. J. Mol. Sci. 18:1576. 10.3390/ijms18071576 28726733 PMC5536064

[B162] Terracciano C. M. N. MacLeod K. T. (1997). Measurements of Ca2+ entry and sarcoplasmic reticulum Ca2+ content during the cardiac cycle in guinea pig and rat ventricular myocytes. Biophys J. 72, 1319–1326. 10.1016/S0006-3495(97)78778-2 9138577 PMC1184514

[B163] Teske B. F. Fusakio M. E. Zhou D. Shan J. McClintick J. N. Kilberg M. S.. (2013). CHOP induces activating transcription factor 5 (ATF5) to trigger apoptosis in response to perturbations in protein homeostasis. Mol. Biol. Cell 24, 2477–2490. 10.1091/mbc.E13-01-0067 23761072 PMC3727939

[B164] Towbin J. A. (2014). Inherited cardiomyopathies. Circ. J. 78, 2347–2356. 10.1253/circj.CJ-14-0893 25186923 PMC4467885

[B165] Tu B. P. Weissman J. S. (2002). The FAD- and O2-dependent reaction cycle of Ero1-mediated oxidative protein folding in the endoplasmic reticulum. Mol. Cell 10, 983–994. 10.1016/S1097-2765(02)00696-2 12453408

[B166] Urbani A. Giorgio V. Carrer A. Franchin C. Arrigoni G. Jiko C.. (2019). Purified F-ATP synthase forms a Ca2+-dependent high-conductance channel matching the mitochondrial permeability transition pore. Nat. Commun. 10:4341. 10.1038/s41467-019-12331-1 31554800 PMC6761146

[B167] Vais H. Mallilankaraman K. Mak D. O. D. Hoff H. Payne R. Tanis J. E.. (2016). EMRE is a matrix Ca2+ sensor that governs gatekeeping of the mitochondrial Ca2+ uniporter. Cell Rep. 14, 403–410. 10.1016/j.celrep.2015.12.054 26774479 PMC4731249

[B168] Vásquez-Trincado C. García-Carvajal I. Pennanen C. Parra V. Hill J. A. Rothermel B. A.. (2016). Mitochondrial dynamics, mitophagy and cardiovascular disease. J. Physiol. 594, 509–525. 10.1113/JP271301 26537557 PMC5341713

[B169] Verfaillie T. Rubio N. Garg A. D. Bultynck G. Rizzuto R. Decuypere J. P.. (2012). PERK is required at the ER-mitochondrial contact sites to convey apoptosis after ROS-based ER stress. Cell Death Differ. 19, 1880–1891. 10.1038/cdd.2012.74 22705852 PMC3469056

[B170] Vincent A. E. Turnbull D. M. Eisner V. Hajnóczky G. Picard M. (2017). Mitochondrial nanotunnels. Trends Cell Biol. 27, 787–799. 10.1016/j.tcb.2017.08.009 28935166 PMC5749270

[B171] Wang G. Hamid T. Keith R. J. Zhou G. Partridge C. R. Xiang X.. (2010). Cardioprotective and antiapoptotic effects of heme oxygenase-1 in the failing heart. Circulation 121, 1912–1925. 10.1161/CIRCULATIONAHA.109.905471 20404253 PMC2917269

[B172] West G. Gullmets J. Virtanen L. Li S. P. Keinänen A. Shimi T.. (2016). Deleterious assembly of the lamin A/C mutant p.S143P causes ER stress in familial dilated cardiomyopathy. J. Cell Sci. 129, 2732–2743. 10.1242/jcs.184150 27235420 PMC4958296

[B173] Wideman J. G. Balacco D. L. Fieblinger T. Richards T. A. (2018). PDZD8 is not the “functional ortholog” of Mmm1, it is a paralog. F1000Research 7:1088. 10.12688/f1000research.15523.1 30109028 PMC6069729

[B174] Wiersma M. Meijering R. A. M. Qi X. Y. Zhang D. Liu T. Hoogstra-Berends F.. (2017). Endoplasmic reticulum stress is associated with autophagy and cardiomyocyte remodeling in experimental and human atrial fibrillation. J. Am. Heart Assoc. 6:e006458. 10.1161/JAHA.117.006458 29066441 PMC5721854

[B175] Wiersma M. van Marion D. M. S. Wüst R. C. I. Houtkooper R. H. Zhang D. Groot N. M. S.. (2019). Mitochondrial dysfunction underlies cardiomyocyte remodeling in experimental and clinical atrial fibrillation. Cells 8:1202. 10.3390/cells8101202 31590355 PMC6829298

[B176] Wozniak M. J. Bola B. Brownhill K. Yang Y. C. Levakova V. Allan V. J. (2009). Role of kinesin-1 and cytoplasmic dynein in endoplasmic reticulum movement in VERO cells. J. Cell Sci. 122, 1979–1989. 10.1242/jcs.041962 19454478 PMC2723153

[B177] Wu S. Lu Q. Ding Y. Wu Y. Qiu Y. Wang P.. (2019). Hyperglycemia-driven inhibition of AMP-activated protein kinase 2 induces diabetic cardiomyopathy by promoting mitochondria-associated endoplasmic reticulum membranes *in vivo*. Circulation 139, 1913–1936. 10.1161/CIRCULATIONAHA.118.033552 30646747 PMC6465113

[B178] Xie W. Santulli G. Reiken S. R. Yuan Q. Osborne B. W. Chen B. X.. (2015). Mitochondrial oxidative stress promotes atrial fibrillation. Sci. Rep. 5:11427. 10.1038/srep11427 26169582 PMC4501003

[B179] Xu H. Guan N. Ren Y. L. Wei Q. J. Tao Y. H. Yang G. S. (2018). IP3R-Grp75-VDAC1-MCU calcium regulation axis antagonists protect podocytes from apoptosis and decrease proteinuria in an Adriamycin nephropathy rat model. BMC Nephrol. 19:140 10.1186/s12882-018-0940-3 29907098 PMC6003198

[B180] Yang F. Yu X. Li T. Wu J. Zhao Y. Liu J.. (2017). Exogenous H2S regulates endoplasmic reticulum-mitochondria cross-talk to inhibit apoptotic pathways in stz-induced type i diabetes. Am. J. Physiol. Endocrinol. Metab. 312, E190–E203. 10.1152/ajpendo.00196.2016 27998959

[B181] Yang Y. Zhang Y. Liu X. Zuo J. Wang K. Liu W.. (2013). Exogenous taurine attenuates mitochondrial oxidative stress and endoplasmic reticulum stress in rat cardiomyocytes. Acta Biochim. Biophys. Sin. 45, 359–367. 10.1093/abbs/gmt034 23619568

[B182] Yaras N. Ugur M. Ozdemir S. Gurdal H. Purali N. Lacampagne A.. (2005). Effects of diabetes on ryanodine receptor Ca release channel (RyR2) and Ca2+ homeostasis in rat heart. Diabetes 54, 3082–3088. 10.2337/diabetes.54.11.3082 16249429

[B183] Yoshida H. Matsui T. Yamamoto A. Okada T. Mori K. (2001). XBP1 mRNA is induced by ATF6 and spliced by IRE1 in response to ER stress to produce a highly active transcription factor. Cell 107, 881–891. 10.1016/S0092-8674(01)00611-0 11779464

[B184] Yu R. Lendahl U. Nistér M. Zhao J. (2020). Regulation of mammalian mitochondrial dynamics: opportunities and challenges. Front. Endocrinol. 11:374. 10.3389/fendo.2020.00374 32595603 PMC7300174

[B185] Yu R. Liu T. Jin S. B. Ning C. Lendahl U. Nistér M.. (2017). MIEF1/2 function as adaptors to recruit Drp1 to mitochondria and regulate the association of Drp1 with Mff. Sci. Rep. 7:880. 10.1038/s41598-017-00853-x 28408736 PMC5429825

[B186] Zhang Z. Zhang L. Zhou L. Lei Y. Zhang Y. Huang C. (2019). Redox signaling and unfolded protein response coordinate cell fate decisions under ER stress. Redox Biol. 25:101047. 10.1016/j.redox.2018.11.005 30470534 PMC6859529

[B187] Zhao Q. Wang J. Levichkin I. V. Stasinopoulos S. Ryan M. T. Hoogenraad N. J. (2002). A mitochondrial specific stress response in mammalian cells. EMBO J. 21, 4411–4419. 10.1093/emboj/cdf445 12198143 PMC126185

[B188] Zhao T. Huang X. Han L. Wang X. Cheng H. Zhao Y.. (2012). Central role of mitofusin 2 in autophagosome-lysosome fusion in cardiomyocytes. J. Biol. Chem. 287, 23615–23625. 10.1074/jbc.M112.379164 22619176 PMC3390636

[B189] Zhao Z. Q. Corvera J. S. Halkos M. E. Kerendi F. Wang N. P. Guyton R. A.. (2003). Inhibition of myocardial injury by ischemic postconditioning during reperfusion: comparison with ischemic preconditioning. Am. J. Physiol. Hear. Circ. Physiol. 285, 579–588. 10.1152/ajpheart.01064.2002 12860564

[B190] Zhu H. Jin Q. Li Y. Ma Q. Wang J. Li D.. (2018). Melatonin protected cardiac microvascular endothelial cells against oxidative stress injury via suppression of IP3R-[Ca2+]c/VDAC-[Ca2+]m axis by activation of MAPK/ERK signaling pathway. Cell Stress Chaperones 23, 101–113. 10.1007/s12192-017-0827-4 28669047 PMC5741585

[B191] Zick M. Rabl R. Reichert A. S. (2009). Cristae formation-linking ultrastructure and function of mitochondria. Biochim. Biophys. Acta Mol. Cell Res. 1793, 5–19. 10.1016/j.bbamcr.2008.06.013 18620004

[B192] Zoccarato F. Cavallini L. Alexandre A. (2004). Respiration-dependent removal of exogenous H2O2 in brain mitochondria inhibition by Ca2+. J. Biol. Chem. 279, 4166–4174. 10.1074/jbc.M308143200 14634020

